# Systematic comparison of sequencing-based spatial transcriptomic methods

**DOI:** 10.1038/s41592-024-02325-3

**Published:** 2024-07-04

**Authors:** Yue You, Yuting Fu, Lanxiang Li, Zhongmin Zhang, Shikai Jia, Shihong Lu, Wenle Ren, Yifang Liu, Yang Xu, Xiaojing Liu, Fuqing Jiang, Guangdun Peng, Abhishek Sampath Kumar, Matthew E. Ritchie, Xiaodong Liu, Luyi Tian

**Affiliations:** 1 Guangzhou National Laboratory, Guangzhou, China; 2 School of Life Sciences, Westlake University, Hangzhou, China; 3 Research Center for Industries of the Future, Westlake University, Hangzhou, China; 4 Westlake Laboratory of Life Sciences and Biomedicine, Hangzhou, China; 5 Westlake Institute for Advanced Study, Hangzhou, China; 6 The Walter and Eliza Hall Institute of Medical Research, Parkville, Victoria Australia; 7 Department of Medical Biology, The University of Melbourne, Parkville, Victoria Australia; 8 Center for Cell Lineage and Development, CAS Key Laboratory of Regenerative Biology, Guangdong Provincial Key Laboratory of Stem Cell and Regenerative Medicine, GIBH-HKU Guangdong-Hong Kong Stem Cell and Regenerative Medicine Research Centre, University of Chinese Academy of Sciences, Guangzhou Institutes of Biomedicine and Health, Chinese Academy of Sciences, Guangzhou, China; 9 Institute for Stem Cell and Regeneration, Chinese Academy of Sciences, Beijing, China; 10 Department of Stem Cell and Regenerative Biology, Harvard University. Stanley Center for Psychiatric Research, Broad Institute of MIT and Harvard, Cambridge, MA USA; 11 GMU-GIBH Joint School of Life Sciences, Guangzhou Medical University, Guangzhou, China

**Keywords:** RNA sequencing, Standards

## Abstract

Recent developments of sequencing-based spatial transcriptomics (sST) have catalyzed important advancements by facilitating transcriptome-scale spatial gene expression measurement. Despite this progress, efforts to comprehensively benchmark different platforms are currently lacking. The extant variability across technologies and datasets poses challenges in formulating standardized evaluation metrics. In this study, we established a collection of reference tissues and regions characterized by well-defined histological architectures, and used them to generate data to compare 11 sST methods. We highlighted molecular diffusion as a variable parameter across different methods and tissues, significantly affecting the effective resolutions. Furthermore, we observed that spatial transcriptomic data demonstrate unique attributes beyond merely adding a spatial axis to single-cell data, including an enhanced ability to capture patterned rare cell states along with specific markers, albeit being influenced by multiple factors including sequencing depth and resolution. Our study assists biologists in sST platform selection, and helps foster a consensus on evaluation standards and establish a framework for future benchmarking efforts that can be used as a gold standard for the development and benchmarking of computational tools for spatial transcriptomic analysis.

## Abstract (web summary)

This analysis presents a systematic comparison of 11 sequencing-based spatial transcriptomics methods using well-characterized references, which offers insights into performance variations in spatial transcriptomic techniques.

## Main

The advent of high-throughput sequencing technologies has revolutionized transcriptomics, providing unparalleled insights into the complexities of gene expression. Single-cell RNA sequencing (scRNA-seq) has been instrumental in dissecting cellular heterogeneity but falls short in capturing the spatial context essential for understanding tissue architecture, cellular interactions and functional state^1,2^. To address this limitation, sST has emerged as a pivotal approach, enabling comprehensive transcriptomic profiling while preserving spatial information within tissues^3,4^.

Despite the rapid advancements in sST technologies, the field is still in its very early stages. The imaging-based spatial transcriptomics has a longer history and a collaborative benchmarking effort has been initiated with the SpaceTX consortium^5^. However, a systematic benchmarking study has not been done for sST. Previous studies have established frameworks for comparing single-cell transcriptomic and epigenomic methods, underscoring the necessity for standardized evaluation criteria and reference tissues for technology validation^6–9^, since simulated single-cell and spatial data may not be reliable^10^. While sST technologies share common features, such as the use of spatial DNA barcodes analogous to cell barcodes in scRNA-seq, the methods diverge notably in aspects such as spatial resolution and the preparation of spatially barcoded oligo arrays^11^. This variability introduces challenges in method selection and complicates the establishment of universal evaluation standards.

In the present study, we address this critical gap by conducting a systematic comparison of 11 sST methods. Using a set of reference tissues, including mouse embryonic eyes, hippocampal regions of the mouse brain and mouse olfactory bulbs we generated cross-platform data for sST benchmarking, referred to as cadasSTre. This dataset enables us to evaluate the performance of each technology in terms of spatial resolution, capture efficiency and molecular diffusion. We updated scPipe^12^ to enable preprocessing and downsampling of sST data, to further minimize variability and facilitate the incorporation of future technologies. Our analyses reveal that data generated from different sST technologies exhibit varying capabilities in downstream applications, such as clustering, region annotation and cell–cell communication. Notably, we also highlighted gene detection biases in sST data.

Our study serves multiple purposes: it (1) guides researchers in the selection of appropriate sST methods for their specific biological questions, (2) establishes a framework for future benchmarking endeavors and (3) contributes to the standardization of evaluation criteria in this rapidly evolving field. Furthermore, our work aims to provide a foundation for the assessment of computational tools designed for spatial transcriptomic data analysis.

## Results

### Benchmarking reference tissues and experimental design

We systematically benchmarked spatial transcriptomics (sST) methods based on distinct spatial indexing strategies, encompassing microarray (probe-based and polyA-based 10X Genomics Visium^13^, DynaSpatial^14^), bead-based approaches (HDST^15^, BMKMANU S1000, Slide-seq V2 (ref. ^16^), Curio Seeker (which is the commercialized version of Slide-seq at Curio Bioscience), Slide-tag^17^), polony- or nanoball-based technologies (Stereo-seq^18^, PIXEL-seq^19^, Salus) and microfluidics (DBiT-seq^20^). Details of each sST method are listed in Tables 1 and 2 and Supplementary Table 1.

**Table 1:** Protocols used in different spatial transcriptomic methods (part 1)

	Stereo-seq	BMKMANU S1000	Visium(polyA)	DBiT-seq
Tissue optimization (preexperiment)	1 Tissue fixation	1 Tissue fixation	1 Tissue fixation	
	None	2 Tissue H&E staining	2 Tissue H&E staining	
	None	3 Tissue brightfield imaging	3 Tissue brightfield imaging	
	2 Permeabilization time course	4 Permeabilization time course	4 Permeabilization time course	
	3 TRITC (tetramethylrhodamine) complementary DNA (cDNA) synthesis	5 Cy3 cDNA synthesis	5 TRITC cDNA synthesis	none
	4 Tissue removal	6 Tissue removal	6 Tissue removal	
	5 Chip TRITC imaging	7 Slide Cy3 imaging	7 Slide TRITC imaging	
Permeabilization time	Brain: 12 min	Brain: 15 min	Brain: 12 min	
	Embryo: 18 min	Embryo: 6 min	Embryo: 6 min	
Spatial expression (formal-experiment)	1 Cryosection on the atereo chip	1 Cryosection on the S1000 gene expression slide	1 Cryosection on the Visium gene expression slide	1 Cryosection on poly-l-lysine coated glass slide
	2 Fixation: methanol for 30 min	2 Fixation: methanol for 30 min	2 Fixation: methanol for 30 min	2 Fixation: formaldehyde 20 min
	3 Tissue single-stranded DNA staining	3 Tissue H&E staining	3 Tissue H&E staining	3 Incubations with ADTs
	4 Tissue fluorescein isothiocyanate imaging	4 Tissue brightfield imaging	4 Tissue brightfield imaging	4 RT with Barcode A Oligo-dT
	5 Tissue permeabilization	5 Tissue permeabilization	5 Tissue permeabilization	5 Ligation with Barcode B
	6 Reverse transcription	6 Reverse transcription	6 Reverse transcription	6 Tissue lysis
	7 cDNA release	7 Second strand synthesis	7 Second strand synthesis	7 Template switch
	8 cDNA amplification	8 cDNA amplification	8 cDNA amplification	8 cDNA amplification
	9 cDNA cleanup and cDNA quality control (QC)	9 cDNA cleanup and cDNA QC	9 cDNA cleanup and cDNA QC	9 cDNA cleanup and cDNA QC
	H&E: adjacent tissue section	H&E: same tissue section	H&E: same tissue section	H&E: adjacent tissue section
Library construction	cDNA was fragmented and amplified, then the products were filtered twice.			
	To construct the sequencing library, cDNA was fragmented, end-repaired and A-tailed. Then the adapter was ligated so that dual index PCR could amplify and distinguish these samples by different index sequences. The distribution of the main peak was between 200 and 600 bp and the libraries were sequenced in the same sequencing run.

**Table 2:** Protocols used in different spatial transcriptomic methods (part 2)

	Salus	DynaSpatial	Slide-seq V2/Curio Bio	Visium(probe-based)
Tissue optimization (preexperiment)	1 Tissue fixation	1 Tissue fixation		1 Collect tissue sections
	2 Tissue H&E staining	2 Tissue H&E staining		2 RNA isolation
	3 Tissue brightfield imaging	3 Tissue brightfield imaging		3 RNA QC by RNA integrity number (RIN ≥ 4)
	4 Permeabilization time course	4 Permeabilization time course	none	
	5 Cys cDNA synthesis	5 Cys cDNA synthesis		
	6 Tissue removal	6 Tissue removal		
	7 Slide Cy3 imaging	7 Slide Cy3 imaging		
Permeabilization time	Brain: 6 min	Brain: 10 min	no permeabilization required	no permeabilization required
	Embryo: 6 min	Embryo: 10 min		
Spatial expression (formal-experiment)	1 Cryosection on the Salus chip	1 Cryosection on the DynaSpatial slide	1 Cryosection on bead arrays	1 Cryosection on the glass slide
	2 Fixation: methanol for 30 min	2 Fixation: methanol for 30 min	2 mRNA hybridization for 15 min	2 Fixation: methanol for 40 min
	3 Tissue H&E staining	3 Tissue H&E staining	3 Reverse transcription	3 Tissue H&E staining
	4 Tissue brightfield imaging	4 Tissue brightfield imaging	4 cDNA release	4 Tissue brightfield imaging
	5 Tissue permeabilization	5 Tissue permeabilization	5 cDNA amplification	5 Probe hybridization and ligation
	6 Reverse transcription	6 Reverse transcription	6 cDNA cleanup and cDNA QC	6 Probe release in CytAssist and probe extension
	7 cDNA release	7 cDNA release		7 Preamplification and cleanup
	8 cDNA amplification	8 cDNA preamplification		
	9 cDNA cleanup and cDNA QC	9 cDNA cycle number determination by quantitative PCR		
		10 cDNA amplification		
		11 cDNA cleanup and cDNA QC		
	H&E: same tissue section	H&E: same tissue section		H&E: same tissue section
Library construction	cDNA was fragmented and amplified, then the products were filtered twice.			1 Cycle number determination by quantitative PCR
	To construct the sequencing library, cDNA was fragmented, end-repaired and A-tailed. Then the adapter was ligated so the dual index PCR could amplify and distinguish these samples by different index sequences. The distribution of the main peak was between 200 and 600 bp and the libraries were sequenced in the same sequencing run.			2 Sample index PCR and cleanup (0.85×)

**Fig. 1:**
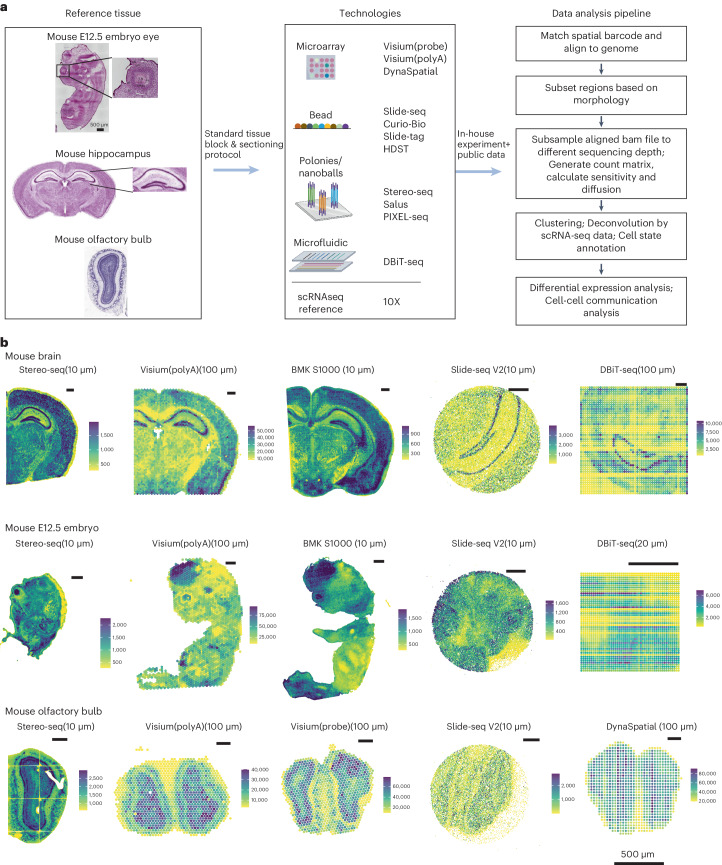
Overview of experimental design and data processing pipeline. **a**, The experimental design involved the use of reference tissues, with technologies categorized by the distinct spatial indexing strategies. **b**, The visualization of total counts across the spatial dimension for datasets generated using each platform for reference tissues is shown. The distances from center to center, used in creating the plot, are presented alongside the name of each sST method. Scale bars, 500 μm.

We selected the adult mouse brain, E12.5 mouse embryo and adult mouse olfactory bulb as reference tissues due to their relatively well-defined morphological characteristics. Adult mouse hippocampus, for instance, exhibits consistent thickness and comprises regions such as cornu ammonis (CA)1, CA2, CA3 and dentate gyrus, each with distinct expression profiles. E12.5 mouse eyes in embryo exhibit a known structure with a lens surrounded by neuronal retina cells, while mouse olfactory bulbs feature clear layer separation with various neuron types. These tissues, with their known morphological patterns and heterogeneous expression profile, serve as ideal reference samples for our sST benchmark studies (Fig. 1a). A summary of the datasets in cadasSTre is given in Supplementary Table 2. Detailed protocols for obtaining regions of interest have been established and are available in the Methods section, facilitating reproducibility by other researchers. We observed highly consistent tissue morphology among different methods in the hematoxylin and eosin (H&E) image, as depicted in Supplementary Figs. 1–5. This validates our standard tissue handling and sectioning procedures in yielding consistent outcomes across diverse experiments. In total, we systematically evaluated 11 sST methods across 35 experiments from three tissue types.

As outlined in the summary pipeline (Fig. 1a, right-hand panel), we next built a standard benchmarking pipeline to enable homogeneous data processing for sST methods and comparison in a fair way. Initially, spatial barcodes and their corresponding locations, together with expression profiles per spatial location, were generated.

Figure 1b and Supplementary Fig. 6 provide an overview of total counts per spot for each sST method across various tissue types. Clear tissue patterns were observed across the samples. The summary of total counts is presented with varying spot sizes and the distances between spot centers. These differences are clearly depicted in Supplementary Fig. 7. In Fig. 1b, we have labeled the distances between spot centers, as we believe this metric better represents the platform’s physical resolution, as opposed to using spot sizes. Stereo-seq, BMKMANU S1000 and Salus have distances between spot centers smaller than 10 μm and spots in them are binned into a 10 μm-sized spots for visualization. We observed that Stereo-seq, Visium, BMKMANU S1000 and Salus managed to capture nearly the entire right brain and the whole E12.5 embryo. Among these methods, Stereo-seq demonstrated the highest capturing capability. Its regular array size is 1 cm and the maximum size goes up to 13.2 cm. In contrast, Slide-seq V2 could capture only a portion of the tissue due to its limited capture size (Supplementary Fig. 7b–d). With DBiT-seq, the capture size varied depending on the width of the microfluidic channel.

Subsequently, we selectively retained reads within regions with known morphology, including the hippocampus in the mouse brain, and eyes in the E12.5 embryo. We then performed downsampling to address sequencing depth and sequencing cost variations. The purpose of downsampling is to normalize different methods to the same total number of sequencing reads to achieve equivalence in sequencing cost. Count matrices with downsampled data and full data were both then generated for sensitivity and diffusion calculations, followed by cell state annotation, maker gene detection and analysis of cell-to-cell communication.

### Molecule-capture efficiency

**Fig. 2:**
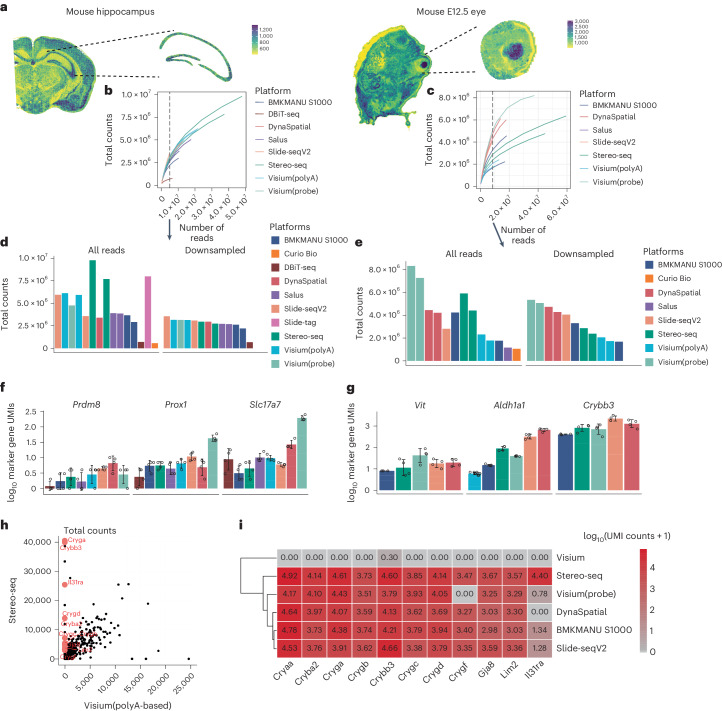
Comparison of the sensitivity of data generated by different platforms. **a**, Schematic plot illustrating the extraction of regions with known morphology from fully processed samples of the adult mouse hippocampus and E12.5 mouse eye. Total UMI counts are presented as a function of stepwise downsampled sequencing depths for each platform. **b**,**c**, The data originate from mouse hippocampus (**b**) and E12.5 mouse eye (**c**) regions. A vertical dashed black line marks the read count used for generating the subsequent downsampled data. **d**, Total UMI counts were computed for selected regions using all reads and downsampled data for the mouse hippocampus. **e**, Total UMI counts for selected regions using all reads and downsampled data for the E12.5 mouse eye. **f**, The summed UMI counts for marker genes across individual 50 × 50 μm regions (*n* = 4) in the mouse hippocampus, along with mean and standard deviation. **g**, The summed UMI counts for marker genes across individual 50 × 50 μm regions (*n* = 4) in the E12.5 mouse eye, along with mean and standard deviation. **h**, Total UMI counts of detected genes are compared between Visium(polyA) (*x* axis) and Stereo-seq (*y* axis). Each dot represents a gene, shown in black. Genes that display expression at the 90th percentile with Stereo-seq but are at the tenth percentile in Visium(polyA) are highlighted in red and labeled with their gene symbols. **i**, A heatmap displays the ${\log }_{10}$-transformed expression of genes that are specifically not captured by Visium(polyA) but are captured by Stereo-seq for E12.5 mouse eyes.

We obtained hippocampus and eye tissues from the adult mouse brain and E12.5 mouse embryo, as illustrated in Fig. 2a. This was accomplished by manually delineating boundaries based on tissue patterns indicated by the spatial distribution of total counts and morphological information provided by H&E images. By selecting the same region, we ensure that our comparisons of sST sample performance were not influenced by varying locations within the tissues, as the number of counts from different parts of the tissue may exhibit variations.

Molecule-capture efficiency was assessed in two ways. In selected regions, we either (1) used all the reads from that region, or (2) downsampled the data so that different samples had the same number of sequenced reads, which we refer to as ‘downsampled data’ in the subsequent results.

Based on the downsampling results (Fig. 2b,c and Supplementary Fig. 8), none of the sequencing runs, which ranged from 300 million reads (Visium) to 4 billion reads (Stereo-seq), reached saturation. This observation suggests that sST data requires considerably more reads for optimal performance, with the potential for increased sensitivity.

Next, we compared the sensitivity of each sST method by summing the total counts within the selected regions. Stereo-seq had many more sequencing reads for the same region compared to other platforms in both mice hippocampus and eye, resulting in relatively higher total counts when all reads are used (Fig. 2d,e, left panel). Despite being nearly half as deeply sequenced as Stereo-seq, Visium(probe) presented the highest summed total counts in mice eye. This might be result of better read-capturing efficiency with the probe-based method and the potential impact of over-quantified unique molecular identifier (UMI) counts when probes are used.

However, when the effect of sequencing depth is controlled, Slide-seq V2 data demonstrated higher sensitivity than other platforms in the mice eye, while in hippocampus, probe-based Visium, DynaSpatial, followed by Slide-seq V2 showed higher sensitivity. This observation aligns with the saturation plot results (Fig. 2b,c), where the summed total count is positively related to the slope at the selected sequencing depth in the saturation plot (Fig. 2d,e, right panel). Additionally, the impact on the relationship between the number of counts and features per spot is more pronounced in Stereo-seq data when comparing downsampled results to the results obtained using all reads (Supplementary Fig. 9).

To provide a more detailed assessment of the differences in sensitivity among selected sST methods, we proceeded to measure the RNA content of marker genes known to be expressed in specific regions using downsampled data. In CA3 of the hippocampus, we compared the sum of counts for *Prdm8*, *Prox1* and *Slc17a7* within 50 × 50 μm regions (selected based on the largest physical resolution value among the sST methods applied). Our findings revealed that the expression patterns of these marker genes somehow mirrored the total count results, with Visium(probe), Slide-seq V2, DynaSpatial exhibiting the highest sensitivity in hippocampus (Fig. 2f). In the case of E12.5 mouse eyes, we compared the sum of counts for *Vit*, *Crybb3* (lens) and *Aldh1a1* (neuron retina) within 50 × 50 μm regions. Similarly, Visium(probe), DynaSpatial, Slide-seq V2, demonstrated the highest sensitivity, while Visium(polyA) did not generate as many counts for marker genes in regions where their expression was expected (Fig. 2g). Through pairwise comparisons, we identified genes consistently expressed in the lens across all sST methods, except for data generated by Visium(polyA) (Fig. 2h and Supplementary Fig. 10a), including *Crybb3* and *Cryaa* (Fig. 2i). This inconsistency did not appear to be attributed to the preprocessing pipeline and gene annotations (Supplementary Fig. 10b), indicating a systematic gene-specific bias of Visium(polyA) toward the lens. In an attempt to correlate this bias with various gene attributes, including gene biotypes, length and guanine-cytosine (GC) content percentage, we discovered that these biased genes, which exhibit low expression in Visium(polyA), are predominantly protein coding. Moreover, no significant bias was detected in terms of GC content or gene length (Supplementary Fig. 11).

In our investigation of the mouse olfactory bulb, after annotation, we assessed the sensitivity of selected sST methods considering layers with varying densities of total counts. Notably, PIXEL-seq exhibited the highest sensitivity, while HDST demonstrated the lowest sensitivity at a 10 μm physical resolution (Supplementary Fig. 12). For methods with resolution at 100 μm, consistent with results in the eye and hippocampus, DynaSpatial and Visium(probe) showed higher sensitivity.

Additionally, we included Slide-tag in the sensitivity comparison, and it displayed the second-highest summed total counts compared to other platforms in mice hippocampus. At the resolution level of 20 μm, Slide-tag generated substantially higher total counts when compared to Stereo-seq and BMKMANU S1000. We observed much better separation of different cell types both in uniform manifold approximation and projection and in marker gene expressions (Supplementary Fig. 13). This shows that the data from other technologies suffer from diffusion and cannot represent a true single-cell expression profile^17^.

### Molecule lateral diffusion

In addition to molecule-capture sensitivity per unit area, another crucial quality parameter is the spatial accuracy of messenger RNA (mRNA) detection. To assess such accuracy, we used two analysis methods to measure molecule lateral diffusion: (1) plotting the intensity profile of a specific gene across the selected region and (2) quantifying the distance between the left-width at half-maximum (LWHM) of intensity in the chosen region^21^, focusing on histological structures in which the expression of the selected gene should exhibit a notable difference, showing high expression in one part of the region and minimal to no expression in the rest. These analyses were conducted using count data generated from all reads.

**Fig. 3:**
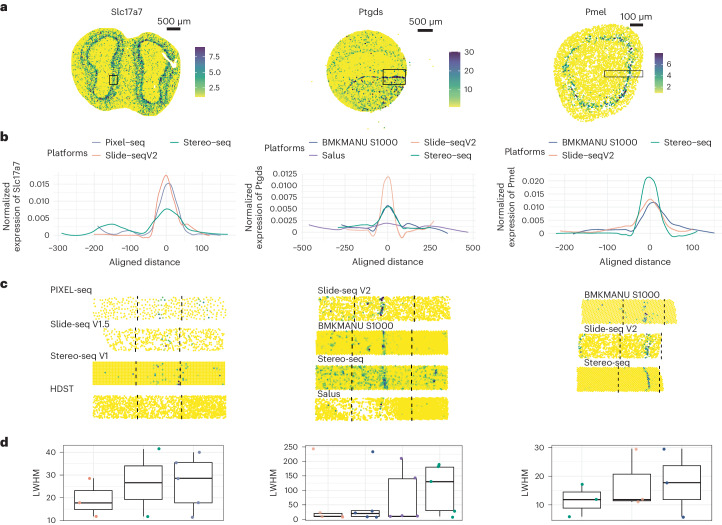
Comparison of diffusion of data generated by different platforms. **a**, Expression markers include *Slc17a7* in the mouse olfactory bulb (left), *Ptgds* in the mouse brain (middle) and *Pmel* in the E12.5 eye (right). The plots are based on raw count values. Black boxes indicate the selected regions used for diffusion calculation. **b**, Expression levels of the aforementioned marker genes (from **a**) are aggregated for every 10 μm along 50 μm in the olfactory bulb, 500 μm in the brain and 300 μm in the eyes, as shown in **a**. UMI counts are averaged across modalities, normalized for each platform and presented in a density plot with the area under the curve set to 1 (details in Methods). **c**, Expression level of the marker genes as mentioned above (from **a**) within selected modalities are provided, with black dashed lines delineating the boundaries used for diffusion calculations. **d**, The left-width at half-maximum (LWHM) of the profile was then calculated for each gene (from **a**) in each modality and displayed in boxplots. Each dot represents the LWHM for a given modality (*n* = 6 for olfactory bulb and brain, *n* = 3 for eye). Modalities for which LWHM could not be calculated were removed. The box denotes the interquartile range, the range between the 25th and 75th percentile, with the median value; whiskers indicate the maximum and minimum value within 1.5 times the interquartile range.

In our evaluation of the olfactory bulb, we selected *Slc17a7* as the marker gene due to its expected expression specifically in mitral and tufted cells, which form distinct layers^22^ and in glutamatergic neurons located at the base of the glomerular layer^23^. We confirmed *Slc17a7*’s expression at these locations via in situ hybridization from the Allen Brain Atlas^24^. In this analysis, our focus was on *Slc17a7*’s expression in mitral and tufted cells. As illustrated by the expression plots of *Slc17a7* in each sST dataset (Fig. 3a, left panel, and Supplementary Fig. 14a), we specifically selected regions (*n* = 6) where *Slc17a7* was expressed in the middle. Our observations, based on intensity plots and LWHM measurements, revealed notable lateral diffusion by Stereo-seq of *Slc17a7* in the olfactory bulb. Notably, Slide-seq V1.5 and PIXEL-seq exhibited relatively better control over this diffusion (Fig. 3b–d, left panel). Methods with lower resolution are compared in a zoom-out view across multiple layers and showed different diffusion performance, with Visium(polyA) preserving more clearly two peaks of the expression of *Slc17a7* across layers in the olfactory bulb, while DynaSpatial barely provided separated expression peaks (Supplementary Fig. 14b,c).

In our analysis of the brain, we selected *Ptgds* as the marker gene, as it has been confirmed by in situ hybridization to be specifically expressed in a particular location within vascular cells^25^ (Supplementary Fig. 15a,b). By examining the expression plots of *Ptgds* and its density plots along with LWHM measurements, we noted severe lateral diffusion in the Stereo-seq dataset. In contrast, Slide-seq V2, followed by BMKMANU S1000, exhibited better control over such lateral diffusion issues (Fig. 3a–d, middle panel, and Supplementary Fig. 15c). We further validated these observations by conducting a diffusion analysis on downsampled Stereo-seq data, confirming that the challenge of lateral diffusion persisted despite a lower sequencing depth compared to other sST datasets. (Supplementary Fig. 15d–f). This suggests that downsampling could not resolve the lateral diffusion issue for Stereo-seq data. Furthermore, we delved into the expression patterns of *Sst*, a gene predominantly expressed in sparsely distributed SST neurons within the mouse cortex^18^. Our analysis found that *Sst* gene expression exhibited more dispersed distribution within each cluster when assessed using Stereo-seq. Larger variance indicated more diffusion assuming the Sst neurons were of similar size in cell body across different samples. This observation corroborates our earlier findings related to diffusion patterns in different technologies (Supplementary Fig. 16).

For our examination of eye tissue, we selected *Pmel* as the marker gene due to its specific expression in melanocytes, which encircles the lens and forms a circular pattern^26^. In this context, Stereo-seq demonstrated the best control over lateral diffusion, followed by Slide-seq V2. (Fig. 3a–d, right panel, and Supplementary Fig. 17). This observation contrasts with our findings in the other two tissue types, indicating that tissue type exerts a considerable influence on the diffusion process. Another factor is permeabilization time, which we showed had a great impact on the diffusion pattern and mRNA capturing (Supplementary Figs. 1–5).

### Clustering and cell annotation across technologies

We next applied selected sST methods to gain insight into biological questions where higher capture sensitivity and well-controlled diffusion are important. We selected E12.5 mice eyes, known for their distinctive structure featuring the lens surrounded by the retina and then melanocytes^27–29^.

#### Annotating regions by clustering results

With the basic knowledge of general cell states within the eye area, our next objective was to annotate the spots captured by selected sST platforms using various clustering methods. We aimed to determine whether we could consistently identify more detailed and coherent cell subsets across all samples.

Such resulting annotations of cell subsets not only served as a benchmark for evaluating the methods used in this study, but also provided valuable insights into the intricacies of cell states within the developing eyes of E12.5 mice.

**Fig. 4:**
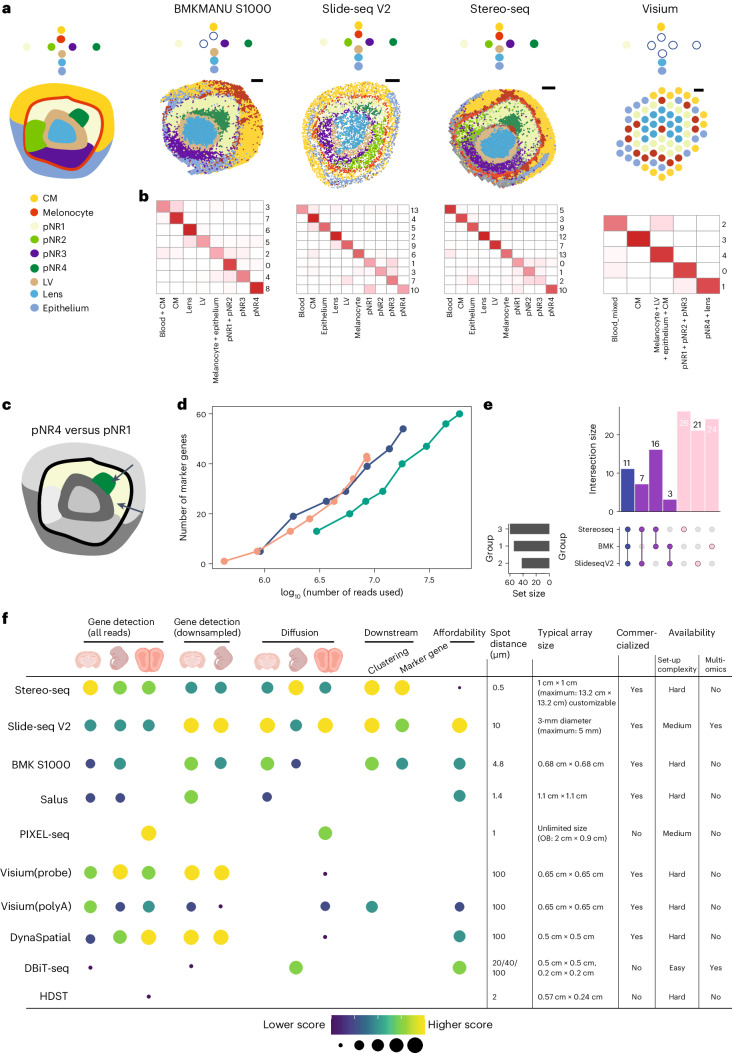
Comparison on downstream performance. **a**, Expression profiles generated by each platform were processed to obtain clustering results. Known cell types and states are colored in the left-most panel. Additionally, a schematic plot represents the expected cell states, arranged from the outer space to inner space and from top to bottom. On the right-hand side, clustering results are presented, with spots color-coded by annotated cell states depicting the identifiable cell states. **b**, Clustering was conducted on downsampled eye data from each platform, with an equal total read count across platforms in the eye area. The correspondence between annotations obtained from clustering based on all reads and clustering based on downsampled data is visualized in a heatmap. The number of spots in this correspondence is presented after ${\log }_{10}$ transformation without scaling. **c**, An overview of cell states compared in the marker gene detection analysis, with pNR4 and pNR1 highlighted. **d**, Number of marker genes detected with different numbers of reads used for each sST method in the comparison between pNR4 and pNR1. **e**, An upset plot displays the intersection of marker genes obtained by different sST methods using all reads for the pNR4 and pNR1 comparison. Genes shared among all three platforms are denoted in blue, those shared between two platforms are in purple and uniquely obtained genes are represented in pink. **f**, The sST methods have been ranked based on their performance in the specified categories, with the highest-performing methods positioned at the top. Methods that offer resolution levels below 20 μm have been given higher preference. In the right panel, essential characteristics of the sST methods examined are outlined. Lower affordability indicates a higher price associated with the method. CM, corneal mesenchyme; pNR, presumptive neural retina; LV, lens vesicle; OB, olfactory bulb.

Before delving into our comparative analyses, Fig. 4a showcases our findings about the cell subsets that we expected to observe within an E12.5 mouse eye. In this tissue, the anticipated morphological structure unfolds from the innermost space, housing the lens and lens vesicle, which are enveloped by neuronal retina cells forming distinct subsets in specific locations. The neuronal retina cells are encircled by melanocytes, with the rostral side hosting the corneal mesenchyme while the caudal side is composed of epithelial cells. These annotations provided us with a foundation for our subsequent evaluations and comparative assessments.

#### Comparison between clustering results

In our comparative analysis of clustering results, we conducted evaluations from two perspectives:

1. Clustering methods: we systematically used three distinct clustering methods: Seurat^30^ (which exclusively considers transcriptomic profiles), DR.SC^31^ and PRECAST^32^ (which incorporate spatial information alongside gene expression data). Recent benchmark studies have reported that methods leveraging spatial location information demonstrate promising clustering results in specific datasets. However, they do not consistently surpass or exhibit greater robustness compared to methods that solely rely on gene expression data^33^. Our observations align with this conclusion, with Seurat consistently demonstrating robust and stable performance compared to the other two methods in detecting expected cell subsets as shown in Fig. 4a, left panel, and Supplementary Fig. 18.
2. sST methods: in our comparisons between sST platforms, we focused primarily on the results generated by Seurat. We annotated spots for each sST method individually (Fig. 4a and Supplementary Fig. 19). Our analyses unveiled variations in the ability of different methods to consistently identify the expected cell subsets. Notably, Slide-seq V2 and Stereo-seq data delivered a nice separation of spots for comprehensive subset annotations, successfully capturing all anticipated subsets. Conversely, BMKMANU S1000 data faced challenges in cell state detection, particularly in identifying melanocytes. This difficulty may stem from the pronounced lateral diffusion observed in BMKMANU S1000 data (as depicted in Fig. 3c,d, right panel), making it difficult for clustering methods relying solely on expression profiles to retain this specific cell type. On the other hand, data with resolution at 100 μm, including Visium and DynaSpatial, faced certain limitations in detecting the anticipated cell subsets. These challenges were primarily attributed to the relatively low physical resolution and hence a restricted number of spots available in the eye area (approximately 75 spots in total for each sample). Within each of these 50 × 50 μm spots, cells were mixed, making identification of intricate cell subsets more challenging (Fig. 4a, right panel, and Supplementary Fig. 19).

#### Influence of downsampling on clustering results

We observed that sequencing depth influences the total counts of spatial transcriptomic data (Fig. 2b–e). In light of this, we set out to investigate how sequencing depth affects clustering results. Our exploration of clustering results on downsampled data involved two key aspects: (1) we assessed the correspondence between the downsampled data and the full data. (2) We calculated entropy measures for cluster purity (ECP) and accuracy (ECA) based on the clustering results obtained with the full data as a reference for downsampled data generated at various proportions as shown in Fig. 4b. We discovered that the downsampled data were capable of detecting nearly all the cell subsets identified by the full data (Fig. 4b and Supplementary Fig. 20). However, when evaluating ECP and ECA across different proportion values, we observed relatively high values, signifying a notable degree of inconsistency. This inconsistency could be attributed to the fact that while most cell subsets effectively formed distinct clusters, a portion of cells grouped into different clusters, notably between cells from different subsets of neuronal retina cells. This effect was particularly pronounced in subsets between populations such as lens and lens vesicles and four neuron retina subsets (Fig. 4b), which are more similar in expression profiles.

#### Comparison between sST data and snRNA-seq data

We consistently observed well-patterned expression of *Pmel*, *Crybb3*, *Atoh7*, *Enfa5*, *Aldh1a1* and *Aldh1a3* across all sST datasets. These genes were selected as they serve as markers for specific cell types, such as melanocytes, lens and presumptive neural retina (pNR)2 and pNR3 (Supplementary Figs. 21 and 22). However, the absolute position for some of the region is not exactly the same but their relative position remains consistent, such as *Aldh1a3* located in rostral region of the retina layer while *Aldh1a1* patterned toward the caudal region (Supplementary Fig. 23).

In addition, we obtained snRNA-seq data with eye region sectioned as input. However, a limited number of cells were found to express the aforementioned genes, and these cells were primarily clustered in the lower corner of the uniform manifold approximation and projection plot (Supplementary Fig. 21e). Unfortunately, we were unable to further categorize this small subset into more detailed subgroups, as was the case with the sST data. We also noted that *Crybb3*’s expression values in the snRNA-seq data were relatively lower than expected. This is in line with our earlier observation that *Crybb3* was not found to be expressed in the eye area captured by Visium(polyA), indicating a potential capture bias associated with polyA-based Visium technology.

While snRNA-seq data may not capture as many cells in the eye area as sST methods do, it serves as a useful reference dataset for annotating the sST data. As illustrated in Supplementary Fig. 24, the integration of snRNA-seq data with sST data using Seurat aided the annotation of sST data. For instance, it improved the annotation of epithelium cells in Stereo-seq data, which had been relatively challenging due to an unknown cluster of cells with mixed expression profiles. This cluster was better resolved using the projection of snRNA-seq epithelium cells (Supplementary Fig. 24a). Additionally, the projection facilitated the separation of melanocytes and epithelium cells in BMKMANU S1000 data (Supplementary Fig. 24b).

Another issue that deserves mention is the susceptibility of sST technologies to blood contamination, which is often introduced during the tissue preparation and sectioning process and is difficult to avoid. We used the *Hba-a1* gene as an example to evaluate the influence of blood contamination in these sST methods. Our findings revealed that Visium, DynaSpatial, followed by BMKMANU S1000, were notably affected by blood contamination, with all Visium and DynaSpatial spots and 70% of BMKMANU S1000 spots expressing *Hba-a1*. In contrast, Stereo-seq data exhibited a relatively similar level of blood contamination compared to snRNA-seq, and Slide-seq V2 had the lowest amount of blood contamination (Supplementary Fig. 25).

### Marker gene detection across technologies

Previous studies have underlined the effectiveness and robustness of using a Wilcoxon rank-sum test when identifying marker genes^34^. We used this test within Seurat to find marker genes between clusters. Analysis of top marker genes reveals technology-specific biases in the selection of these markers. For instance, *Pax6*, a transcription factor known as a master regulator of neural lineages, particularly in the retina^35^, exhibited variations in representation among different technologies. Specifically, Stereo-seq data highlighted *Pax6* exclusively in the pNR3 cluster, whereas Slide-seq V2 and BMKMANU S1000 data depicted *Pax6* expression across the entire neural retina (pNR1–4) (Supplementary Table 3), consistent with existing literature. This observation underscores the influence of technology choice on the identification of top markers for specific cell types or clusters. Similarly, disparities were observed in the expression of Hes genes in progenitors of the neural retina and *Sox2* in pNR1 and pNR2 (Supplementary Table 3).

The analysis of clustering results on downsampled data has shown that general cell subsets can still be adequately retained even with fewer sequencing reads. However, it appears that a few subsets, particularly those sharing similar expression profiles, are a challenge to clearly separate. To further investigate the effects of downsampling, we compared the marker genes identified in downsampled data with those in the full dataset. We selected two pairs of cell subsets to compare the detection performance for cell subsets with relatively similar expression profiles and those that are more distinct. We conducted the marker gene detection in two scenarios to identify marker genes: (1) pNR4 and pNR1, which exhibit higher similarity, and (2) lens and melanocytes, which have lower similarity (Fig. 4c and Supplementary Fig. 26a).

Our observations revealed that the number of marker genes increased as the number of reads increased in both pairs of comparisons. Notably, the increase in marker genes was more pronounced with deeper sequencing, as illustrated in Fig. 4d and Supplementary Fig. 26a. The ranking of marker gene detection performance across sST methods aligns with the results depicted in Fig. 2d in the comparison between cell subsets with relatively distinct expression profiles. In particular, Slide-seq V2 exhibits higher sensitivity (Supplementary Fig. 26b). Furthermore, our analysis identified each platform have more unique marker genes than shared across technologies (Fig. 4e and Supplementary Fig. 26c).

Cell-to-cell communication was applied afterward, but no consistent results could be found across the communication methods applied including CellChat^36^, CellPhoneDB v.4 (ref. ^37^) and sST methods (Supplementary Fig. 27).

## Discussion

Evaluating spatial transcriptomic methods is more challenging than evaluating scRNA-seq methods. First, it is harder to design a reference tissue for spatial transcriptomics. If we use genuine tissues with clear cell type and gene expression patterns, the position and ground truth become less obvious and limited by our understanding of reference tissues, unlike for scRNA-seq where one could use cell line mixtures or peripheral blood mononuclear cell samples to obtain consistent inputs^8,38,39^. Second, the measurements are not performed on the same unit. For methods such as Visium (both polyA-based and probe-based) and DynaSpatial, the diameter of a spot is larger than 50 μm resembling a mini-bulk RNA-seq. For methods such as Stereo-seq, the spot size is submicrometer, which is much smaller than a single cell.

We carefully designed our benchmarking study to address these challenges. We selected a set of reference tissues with the following criteria: (1) the tissue should be from the widely used model organism, accessible at most research institutes; (2) the tissue should have stable cell-type patterns and specific marker gene expression and (3) the reference region should have clear morphology that is easy to find in sectioning. Together with the reference tissue, we developed a sectioning protocol to help people reproduce and generate comparable data in the future. For the second challenge, we used multiple benchmarking metrics and workflows to compare different methods on the same tissue region. We used both all reads and downsampled data in our comparisons. Downsampling was implemented to mitigate the impact of variations on sequencing depth and cost, however, as this may not bring all methods to the same standard because the required number of reads to achieve satisfactory results may differ, we also used all reads in the analysis as complementary results.

In this study, we generated cadasSTre at genographix.com, a cross-platform dataset for sST benchmarking that allowed systematic evaluation of 11 sST methods across 35 experiments. We compared various aspects of data from basic metrics to downstream analysis, ranging from sensitivity and diffusion to clusterability and marker gene detection (Fig. 4f). A summary of the methods employed in each evaluation step can be found in Supplementary Table 4. Our results indicate spatial transcriptomics requires more sequencing to reach saturation and data generated in this study are well below the saturation level. Stereo-seq, Slide-tag, Visium(probe) shows the better capture efficiency with raw sequencing depth while Slide-seq V2, Visium(probe), DynaSpatial gives the better capture efficiency with normalized sequencing depth. We found unexpected gene-capturing bias on the polyA-based Visium platform, with marker genes consistently captured by other technologies not showing up in the Visium(polyA) data. Considering Visium is the most widely used commercial platform, it is important to further verify its gene-capturing bias on other tissues.

The spot size has become an important metric as a surrogate of the resolution for each method. However, in this study, we highlighted diffusion as a key factor that affects the actual resolution. Our permeabilization optimization experiment, as depicted in Supplementary Figs. 1–5, demonstrates that varying the permeabilization time has a substantial impact on diffusion. On optimizing the permeabilization time for each tissue type, different technologies exhibited varied diffusion profiles across various tissue types. Although some technologies have subcellular spot sizes, their real resolution would not reach the same level due to limited sensitivity and high diffusion compared to Slide-tag that has true single-cell resolution. Further development of sST would benefit from increased diffusion control and improved assay to determine the permeabilization condition and time.

Although the goal of this study is not to comprehensively benchmark computational tools, we found that clustering tools designed for spatial data may not give better performance than clustering methods for single cells, which agrees with a comparison study^33^. We also found that cell annotations derived from single-cell references may not yield detailed cell states and that clustering derived from spatial data could give complementary results that were sometimes better at resolving rare cell states with spatial patterns. It is important to consider both analyses with and without single-cell references when annotating spatial data.

Overall, our study generated the first systematic benchmarking scheme of sST methods. Although we strive to show the best of all the technologies, some of the experiments are not fully optimized, such as DBiT-seq and Curio Bio, which means their data may not represent their best performance. sST experiments generally requires more experimental skills and operational effects cannot be neglected. The sST field is rapidly evolving and the performance of each technology is likely to change with time as they are further optimized. Continuing evaluation is required to keep pace with this fast-moving field. Spatial multi-omics methods are still in their early stages of development^40–43^, and new technologies need to be established. Therefore, we will continue to maintain and update our online benchmarking database with new datasets, all hosted at genographix.com. Through this platform, researchers can access most updated benchmarking data, contribute their findings and collaborate with peers to advance the field.

## Methods

### Sample preparation

#### Reference sample

All relevant procedures involving animal experiments presented in this study are compliant with ethical regulations regarding animal research and were conducted under the approval of the Animal Care and Use Committee of Westlake University (license number AP#23-111-LXD). Animals were group housed with a 12 h light–dark schedule and allowed to acclimate to their housing environment for 2 weeks postarrival. Mouse embryos were collected from pregnant C57BL/6J female mice at embryonic day 12.5 (E12.5). Mouse brain was dissected from 8 week-old C57BL/6J male mice.

#### Sample preparation, embedding, sectioning and histological testing

##### Mouse embryo

E12.5 pregnant female mice were anesthetized with carbon dioxide, and the whole uterus was collected and washed three times in ice-cold DPBS. The uterus was separated under a stereo microscope, and each embryo was numbered and photographed with a Motorized Fluorescence Stereo Zoom microscope (Zeiss, Axio Zoom V16). A yolk sac was collected to extract DNA for genotyping (identification of sex). Using dust-free paper to gently wipe the liquid on the surface of the embryo, the embryo was rinsed with ice-cold Tissue-Tek optimal cutting temperature (OCT) (Sakura, cat. no. 4583), and then moved to the encapsulation box with ice-cold OCT. Air bubbles were carefully removed with the syringe, and the embryo was placed in the sagittal position with tweezers. The location of the embryonic eye was circled and marked the orientation of the embryo, then tissues were transferred to a −80 °C freezer, snap-frozen and stored. Embryos of average size and normal phenotype were selected for subsequent cryosectioning and sequencing (note that the embryos used in our benchmarking analysis came from a litter of mice). Before sectioning, the tissue block was removed from the −80 °C freezer and placed in a cryostat (Leica, cat. no. CM1950) to balance for at least 30 min. Then, the tissue block was smoothly glued to the sample head so that the embryo was sectioned in a sagittal position. If necessary, the angle can be fine-tuned so that the blade section is strictly parallel to the cross-section of the tissue block. Cryosections were cut at a thickness of 10 μm, both the left eye and right eye can be collected. The structure of the sequenced cryosections is shown in Supplementary Fig. 28a.

The H&E staining procedure was as follows: cryosections were balanced at room temperature for 30 min, and then fixed with 4% PFA for 3 min. Then, the sections were washed with ddH_2_O for 2 min, stained with hematoxylin for 6 min, washed with ddH_2_O, stained with eosin for 2 min and washed with ddH_2_O. After that, sections were gradient dehydrated (75% ethyl alcohol for 1 s, 85% ethyl alcohol for 1 s, 95% ethyl alcohol for 1 s, 100% ethyl alcohol for 1 s, 100% ethyl alcohol for 1 min), cleared (xylene for twice) and sealed with Permount TM Mounting Medium after airing. Finally, the figure was scanned using a Motorized Fluorescence Microscope (Nikon, Ni-E).

##### Mouse brain

Eight-week-old male mice were anesthetized with carbon dioxide and decapitated. The whole brain was rapidly dissected, numbered and photographed with a Motorized Fluorescence Stereo Zoom microscope. Using dust-free paper to gently wipe the liquid on the surface of the brain, the brain was rinsed with ice-cold Tissue-Tek OCT (Sakura, cat. no. 4583), and then moved to an encapsulation box with ice-cold OCT. Air bubbles were carefully removed with the syringe, and the brain was placed properly with tweezers. The location of the hippocampus was circled and marked the orientation of the brain, then tissues were transferred to a −80 °C freezer for snap-freezing and storage. Brains of average size and normal phenotype were selected for subsequent cryosection and sequencing. Before sectioning, the tissue block was taken out from the −80 °C freezer and placed in a cryostat (Leica, cat. no. CM1950) to balance for at least 1 h. Then, the tissue block was smoothly glued to the sample head, and the cerebellum was oriented toward the experimenter so that the brain was sectioned in a coronal position. If necessary, the angle could be fine-tuned so that the blade section was strictly parallel to the cross-section of the tissue block. Cryosections were cut at a thickness of 10 μm. The structure of the sequenced cryosections is shown in Supplementary Fig. 28b.

H&E staining procedure: cryosections were balanced at room temperature for 30 min, and then fixed with 4% PFA for 3 min. Then, the sections were washed with ddH_2_O for 2 min, stained with hematoxylin for 6 min, washed with ddH_2_O, stained with eosin for 1 min and washed with ddH_2_O. After that, sections were gradient dehydrated (75% ethyl alcohol for 1 s, 85% ethyl alcohol for 1 s, 95% ethyl alcohol for 1 s, 100% ethyl alcohol for 1 s, 100% ethyl alcohol for 1 min), cleared (xylene twice) and sealed after airing. Finally, the figure was scanned using a Motorized Fluorescence Microscope (Nikon, Ni-E).

#### In situ imaging with padlock probes

To validate the expression of marker genes, we performed in situ hybridization and imaging following a simplified version of targeted ExSeq^44^. More specifically. we used four fixed barcode regions for distinct fluorescent probes (FAM6, CY3, TXRED, CY5) so we could detect at most four genes at the same time without performing multi-round imaging for in situ sequencing. The tissue was sectioned on Leica CM1950 Cryostats, with 10 μm sections placed on the CITOTEST adhesion microscope slides. The section was then fixed with 4% formalin for 15 min at room temperature and washed twice with PBS. Permeabilization of tissue was done with ice-cold 70% EtOH overnight at −20 °C. RNase inhibitor (Lucigen) was added at 0.4 U μl^−1^ throughout the incubation until the rolling cycle amplification was done. The padlock probe was diluted at a final concentration of 5 nM per probe in wash buffers with 2× SSC and 20% formamide. Hybridization was done overnight at 37 °C, then washed with the same wash buffer (2× SSC and 20% formamide) three times for 15 min each, followed by washing with PBS for 15 min at 37 °C. SplintR ligase (NEB, M0375) was used for probe ligation at 37 °C for 2.5 h. Rolling cycle amplification was performed at 30 °C overnight using Phi29 enzyme mix (NEB, cat. no. M0269L). Fluorescent probe hybridization was done with 2× SSC and 10% formamide buffer mix, diluting the probes at 100 μM and incubating at 37 °C for 1 h. Imaging was performed with a NIKON A1 confocal microscope with ×10 objectives and 2 × 2 image stitching.

#### Protocols used in different spatial transcriptomic methods

Protocols used in different transcriptomics methods are detailed in Tables 1 and 2.

### Data processing

#### Preprocessing

We preprocessed fastq files from multiple platforms using their respective preprocessing pipeline (where provided) and updated scPipe to allow sample processing with unified functions for data from different sST technologies starting from fastq files. Mouse GRCm39 was used as a reference for alignment in each of the pipelines for locally generated data. Visium data were processed with spaceranger (v.2.1.0), and aligned with STAR v.2.7.10b. BMKMANU S1000 is a technology developed by BMKGENE. Similar to HDST, it uses barcoded beads deposited on patterned array. Data were processed with BSTMatrix (v.2.3.j), and aligned with STAR v.2.7.10b. Slide-seq V2 generated bam files of pucks of mouse eyes (Puck_190926_03) and hippocampus (Puck_191204_01 and Puck_200115_08) were downloaded^16^. Stereo-seq data were processed with SAW (v.6.1). DBiT-seq data underwent initial filtering using a predefined barcode list and, subsequently, fastq file 1 was restructured to adopt the format of spatial barcodes followed by UMIs. The processed data were further analyzed using scPipe (v.2.0.0) to generate spot-by-gene count matrices.

#### Selection of region of interest and downsampling

After acquiring count matrices and associated location data for the datasets generated by the aforementioned sST platforms, we aimed to mitigate the impact of variable sequencing depths and costs. To achieve this, we extracted spots located within consensus regions in reference tissues, specifically the hippocampus in the brain and the eye in mouse embryos, for comparative analysis.

Spot selection was guided by histological images (H&E images), feature plots of total counts and marker genes (*Pmel* for the eye and *Slc17a7* for the brain). These boundaries were meticulously delineated manually.

Subsequently, spots falling within the predefined boundaries for each sample were isolated and used for downsampling. Equal numbers of reads were chosen within selected spots for both eye and hippocampus data, based on the readID of the selected reads. These selected reads were then isolated from the BAM files generated in the aforementioned pipelines.

The BAM files were further processed to demultiplex based on spatial barcodes and quantified into matrices using UMIs and aligned gene information, using new functions introduced in scPipe. In addition to generating count matrices with an equivalent number of reads across platforms, we also processed reads from each platform in specific proportions using scPipe.

#### Sensitivity and diffusion of marker genes

For each sample, we calculated the sum of total counts within selected regions using both the full set of reads and downsampled reads. To assess marker gene sensitivity, we considered specific genes known to be expressed in the dorsal anterior region of the hippocampus in adult mice (*Prdm8*, *Prox1* and *Slc17a7*), as well as genes known to be expressed in the lens of the eyes (*Vit* and *Crybb3*) and a subset of neural retina cells in the eyes of E12.5 mice (*Aldh1a1*). In each sample, we selected five regions measuring 50 by 50 μm in the eyes and four regions measuring 50 by 50 μm in the hippocampus, where these genes were known to be expressed. We individually summed the total number of UMIs in these selected regions within downsampled count matrices to ensure the number of reads was consistent across platforms.

We then performed pairwise comparisons of UMI counts for detected genes across platforms. For eye samples, genes expressed in any one of the platforms with total counts above the 99th percentile but below the tenth percentile in any other platforms were selected for heatmap plotting using a log_10_ scale.

To investigate the observed gene bias within the Visium(polyA) dataset, as indicated by the pairwise comparisons with other platforms, we conducted a analysis of variance (ANOVA) test. In the first step, we identified genes expressed across all platforms, ensuring that their total counts exceeded the 90th percentile threshold. Within this selected gene set, we further stratified them based on their expression levels in the Visium(polyA) dataset. Genes exhibiting an expression level below 30 were categorized as bias genes, signifying their lower expression specific to the Visium(polyA) dataset. The remaining genes were used for comparative purposes. Our exploration of these genes encompassed an in-depth analysis of their attributes, including GC content percentage and gene length, using ANOVA analysis. Additionally, we examined the biotypes of these biased genes.

To assess the spatial distribution of marker genes known to be expressed in specific regions of the reference tissues, we used *Pmel* for eye data, *Ptgds* for brain data and *Slc17a7* for olfactory bulb data. This analysis used count matrices generated from all reads. We selected regions with expression of these genes roughly in the middle of the chosen regions (six modalities of 300 by 50 μm in the olfactory bulb, six modalities of 500 by 50 μm in the brain and three modalities of 300 by 50 μm in the eyes). We summed the expression of the aforementioned marker genes for every ten along 50 μm within 300 μm in the olfactory bulb, 500 μm in the brain and 300 μm in the eyes. The UMI counts of these marker genes were then aligned based on the location of peak expression and averaged. Modalities with insufficient counts for the selected marker genes were filtered out before plotting.

After the computation of averaged summed values across modalities, these values were subsequently normalized for each platform and depicted in a density plot with the area under the curve standardized to 1. Subsequently, the left half-width half-maximum (LWHM) of the profile was computed within each modality using nonnormalized expression values across platforms. It is worth noting that we used LWHM as an evaluation metric, drawing inspiration from the full-width at half-maximum method^21^. In the chosen region, only the LWHM was used as there could be an expression of selected genes, such as *Slc17a7* in the olfactory bulb, on the right side of the section that is biologically expected but not caused by lateral diffusion. Modalities that could not be computed with LWHM were excluded from the plotting process. For diffusion analysis, it is important to note that the DBiT-seq data pertained to E10 embryonic eyes, whereas the other datasets were associated with E12.5 embryonic eyes.

To address the notable diffusion in data generated by Stereo-seq in the mouse brain, we conducted diffusion analysis on its downsampled data, consisting of 14% of all the reads.

To analyze the spatial distribution of SST neurons, we operated under the assumption that SST neurons are primarily solitary entities. Our approach involved using the dbscan algorithm to identify clusters of neurons based on the expression of *Sst*. Once isolated clusters were identified, we computed both the average count values and the variance in distances within these clusters. Higher variance values signify counts originating from dispersed locations, which in turn may suggest potential diffusion.

#### Cell-type annotation

Low-quality spots with total counts below 30% of the first quantile of total counts are filtered out before normalization, which was carried out using the median number of total counts from each platform as the scaling factor. Subsequently, the top 2,000 highly variable genes were identified using the FindVariableFeatures function and used to scale the data through the ScaleData function. A total of 20 principal components were then calculated using RunPCA. To categorize spots in each eye sample, we used three distinct methods, including Seurat (v.4.3.0), DR.SC (v.3.3) and PRECAST (v.1.6.2).

Seurat initially identified neighbors based on 20 principal components, with a *k* value of 5 chosen for the *k*-nearest neighbor algorithm in FindNeighbors. FindClusters was subsequently applied with various physical resolutions to group known cell-type spots. DR.SC was applied by setting *K* (the number of clusters) to ten. PRECAST was applied with the number of clusters specified as ten and using the SelectModel function to reorganize the fitting results within PRECASTObj.

#### Integration of sST and scRNA-seq data

We followed the instructions in Seurat with parameters reduction =‘cca’, k.filter = NA and normalization.method = ‘SCT’ in FindTransferAnchors. Dimensions were set to 30 with principal components analysis used as weight.reduction in TransferData.

#### Evaluation of clustering on downsampled data

The downsampled data were subjected to the same pipeline as described above, leveraging Seurat to generate clustering results. These obtained clustering results were subsequently compared to clustering outcomes obtained through the processing of count matrices generated from the entire set of reads. To visualize this comparison, we created a heatmap with a logarithmic scale, illustrating the corresponding number of spots in each of the downsampled clustering and the overall clustering results. The entropy of accuracy (ECA) and purity (ECP) were then calculated. ECA and ECP are defined as follows:

1 $${\mathrm{ECA}}=-\frac{\mathop{\sum }\nolimits_{i = 1}^{M}\mathop{\sum }\nolimits_{j = 1}^{{N}_{i}}P({x}_{j})\log (P({x}_{j}))}{M}$$

where *M* denotes the number of clusters generated from a method (the clustering solution to be evaluated) and *N*_*i*_ denotes the number of elements in the *i*th cluster based on the ground truth (here the provided labels) and

2 $${\mathrm{ECP}}=-\frac{\mathop{\sum }\nolimits_{i = 1}^{N}\mathop{\sum }\nolimits_{j = 1}^{{M}_{i}}P({x}_{j})\log (P({x}_{j}))}{N}.$$

#### Marker genes detection

FindMarkers in Seurat was applied to find marker genes between two pairs of cell subsets: (1) lens and melanocytes and (2) pNR4 and pNR1. Genes that exhibited higher expression in the lens and pNR4, with expression levels exceeding 5% of the specific spots, a log fold change greater than 0.25 and an adjusted *P* value provided by Seurat less than 0.01, were considered to be marker genes.

#### Cell communication analysis

Cell communication analysis was then performed on spots with distinct annotated cell types using methods including Cellchat (v.1.6.1) and CellPhoneDB (v.4). Cellchat used the CellChatDB database of the mouse, creating Cellchat objects based on annotation information, and employed the default ‘Trimean’ statistical method. CellPhoneDB was applied by transforming mouse genes into their human homologs using the biomaRt package. Using the CellPhoneDB database for cellphone analysis, we conducted using 1,000 random permutations in the analysis following the tutorial https://github.com/ventolab/CellphoneDB/blob/master/notebooks/T01_Method2.ipynb. The minimum cell percentage threshold required to consider a gene as expressed in the analysis was set to 0.1, and significance was determined with a *P v*alue threshold of less than 0.05.

### Reporting summary

Further information on research design is available in the Nature Portfolio Reporting Summary linked to this article.

## Online content

Any methods, additional references, Nature Portfolio reporting summaries, source data, extended data, supplementary information, acknowledgements, peer review information; details of author contributions and competing interests; and statements of data and code availability are available at 10.1038/s41592-024-02325-3.

## Supplementary information

Supplementary Information Supplementary Figs. 1–28.

Reporting Summary

Supplementary Table 1 Summary of applied sST methods.

Supplementary Table 2 Summary of datasets used in the study.

Supplementary Table 3 Summary of differentially expressed genes obtained in the marker gene detection analysis.

Supplementary Table 4 Summary of downstream analysis applied for each dataset in the comparisons.

## Data Availability

Raw count matrices are available at the National Genome Data Center (https://www.cncb.ac.cn/) under BioProject accession code PRJCA020621. A summary of individual accession numbers is given in Supplementary Table 2. The cadasSTre data collections are continually updated on our website genographix.com. The standard sectioning protocol is deposited in protocols.io: 10.17504/protocols.io.5qpvo379dv4o/v1.

## Code availability

Scripts used to process the data are available via GitHub at https://github.com/YOU-k/cadasSTre.
