# Supplementary material for: Systematic comparison of sequencing-based spatial transcriptomic methods
Source: Nat Methods. 2024 Jul 4;21(9):1743–54. doi: 10.1038/s41592-024-02325-3 (PMC11399101; doi:10.1038/s41592-024-02325-3)
Supplement: Supplementary file 1 — Supplementary Figs. 1–28. [file 41592_2024_2325_MOESM1_ESM.pdf]

nature methods

Supplementary information https://doi.org/10.1038/s41592-024-02325-3

Systematic comparison of sequencing-based spatial transcriptomic methods

In the format provided by the authors and unedited

10X Visium (polyA)

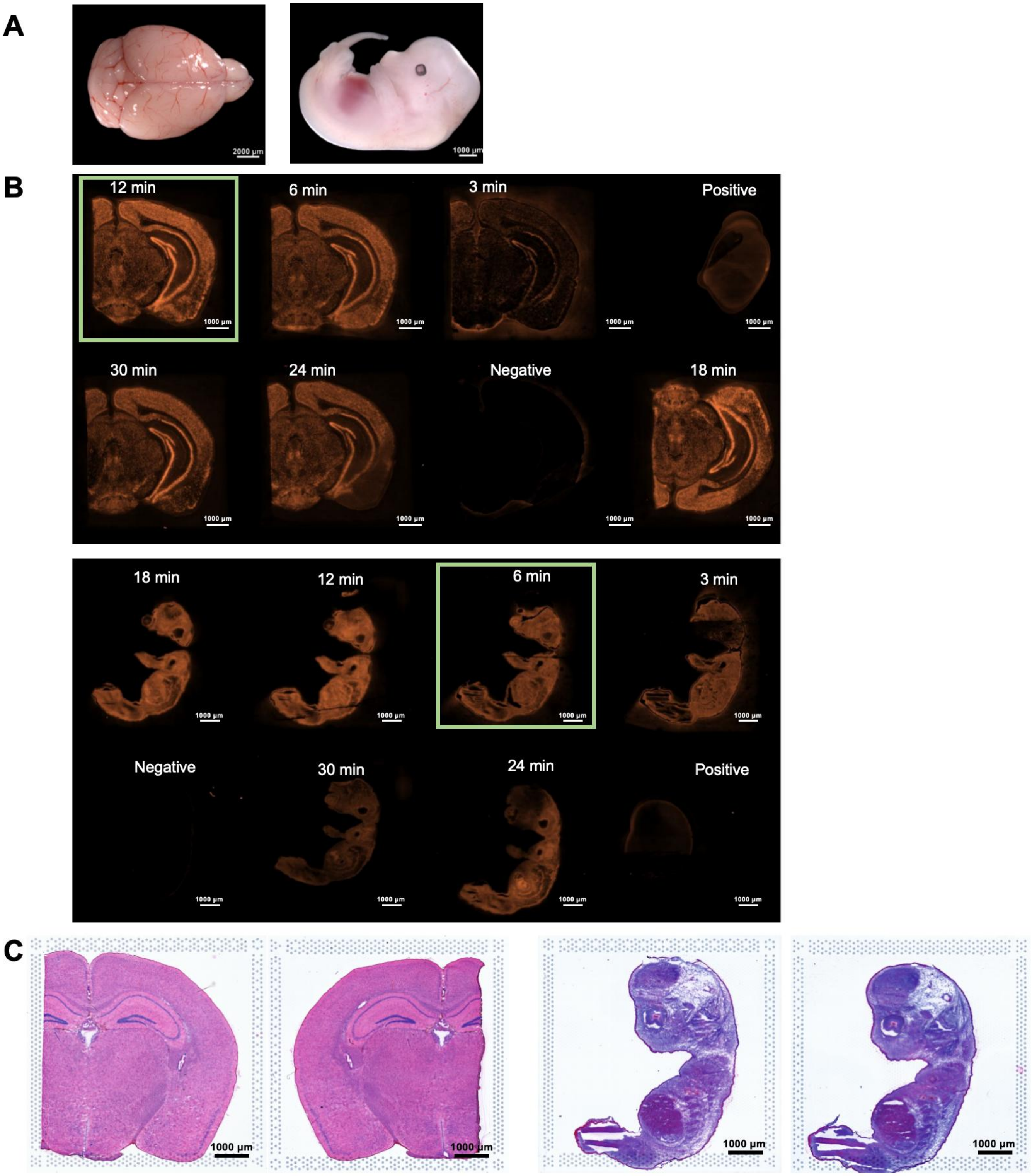

Supplementary Fig.1.
A. Tissue blocks used for 10X Visium (polyA) spatial technologies.
B.Tissue permeabilization were optimized before spatial gene expression experiment. Permeabilization for 3 min, 6 min, 12 min, 18 min, 24 min, and 30 min were performed, and fluorescence value was used for evaluation. 12 min, 6 min permeabilization time was chosen for the spatial gene expression experiment of mouse brain and E12.5 embryonic eye, respectively. No replicate was applied.
C. H&E staining of sections used for spatial expression.

Stereo-seq

A

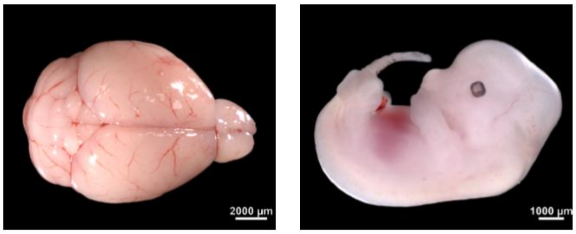

B

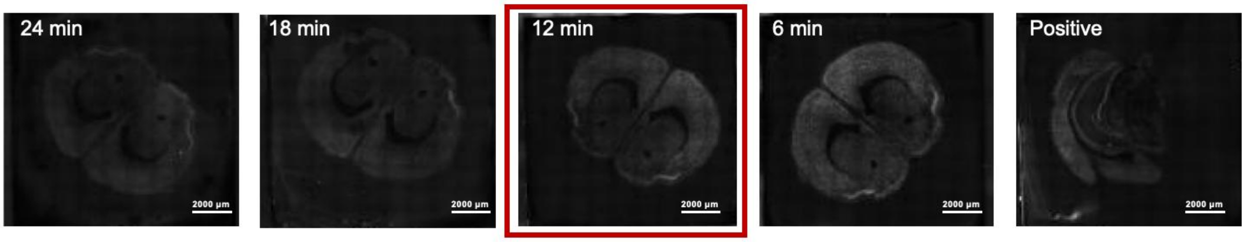

permeabilization time	24 min	18 min	12 min	6 min	Positive
gray value	22.08	25.651	25.163	28.008	-

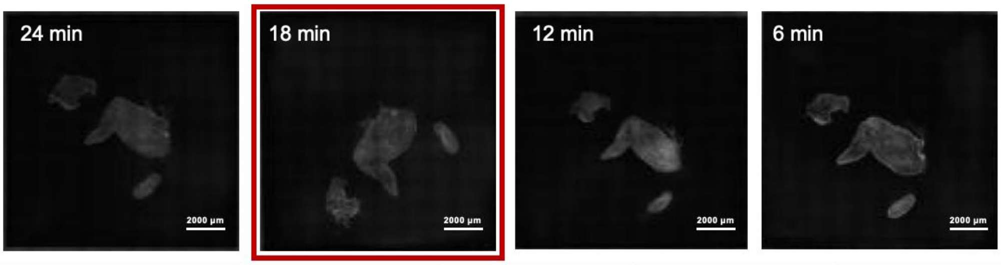

permeabilization time	24 min	18 min	12 min	6 min
gray value	13.074	18.804	12.893	15.399

C

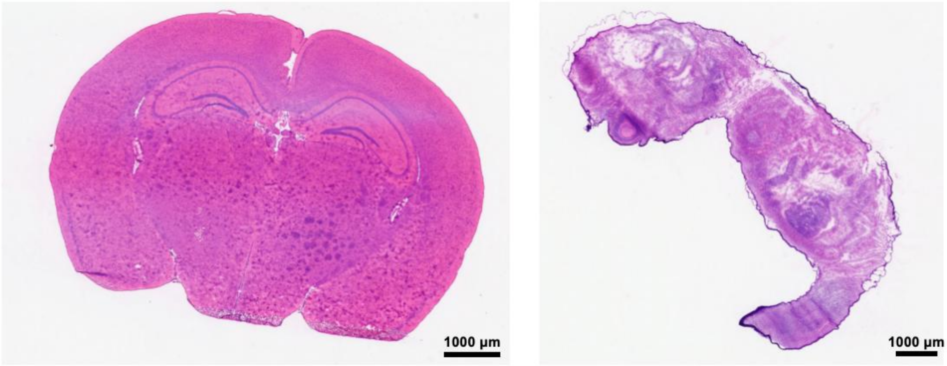

Supplementary Fig.2.
A. Tissue blocks used for Stereo-seq spatial technologies.
B.Tissue permeabilization were optimized before spatial gene expression experiment. Permeabilization for 6 min, 12 min, 18 min, and 24 min were performed, and fluorescence value was used for evaluation. 12 min, 18 min permeabilization time was chosen for the spatial gene expression experiment of mouse brain and E12.5 embryonic eye, respectively. No replicate was applied.
C. H&E staining of adjacent sections used for spatial expression.

BMK S1000

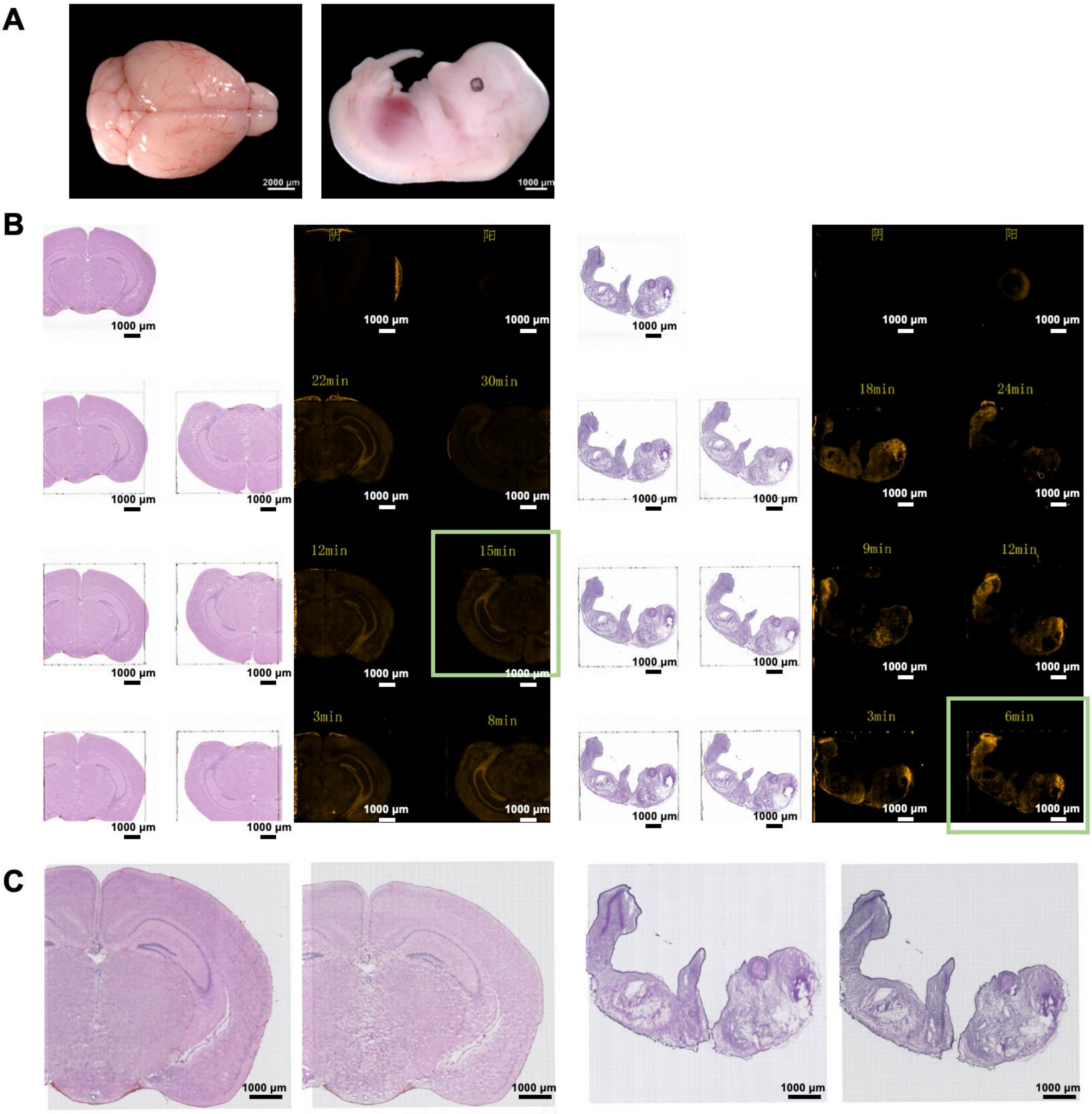

Supplementary Fig.3.
A. Tissue blocks used for BMK S1000 spatial technologies.
B.Tissue permeabilization were optimized before spatial gene expression experiment. Permeabilization for 3 min, 8 min, 12 min, 15 min, 22 min, and 30 min were performed, and fluorescence value and corresponding H&E staining was used for evaluation. 15 min, 6 min permeabilization time was chosen for the spatial gene expression experiment of mouse brain and E12.5 embryonic eye, respectively. No replicate was applied.
C. H&E staining of sections used for spatial expression.

DynaSpatial

a

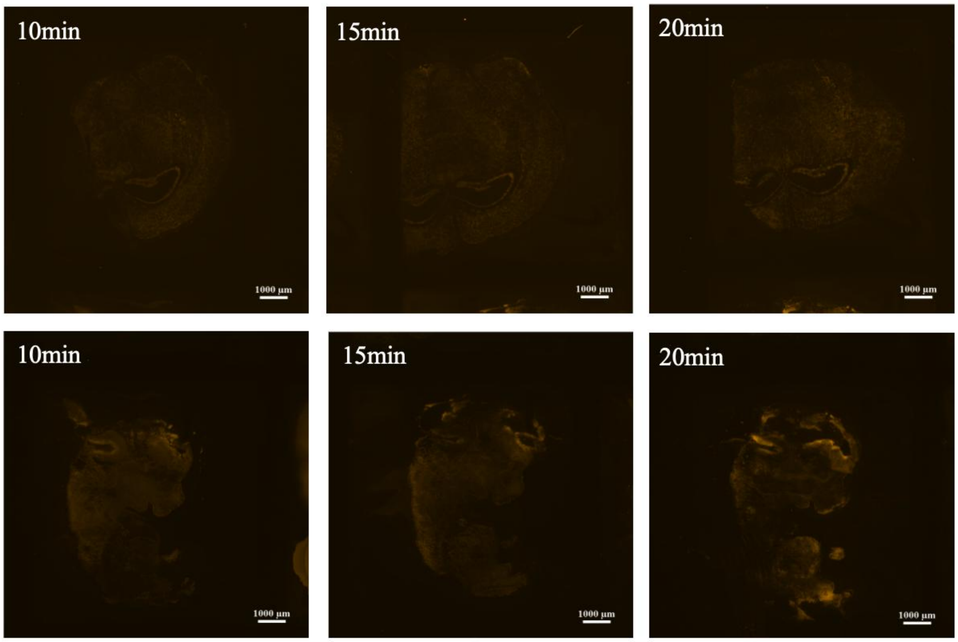

b

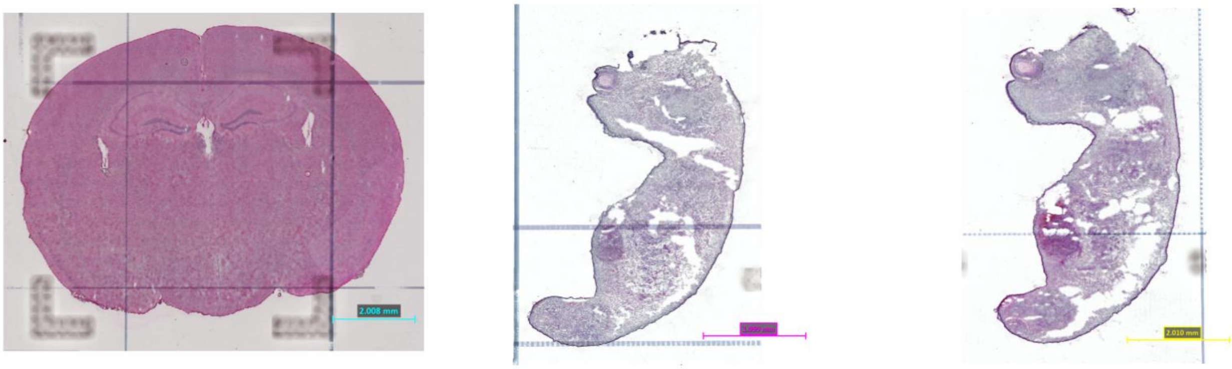

Supplementary Fig.4.

A. Tissue permeabilization were optimized before spatial gene expression experiment. Permeabilization for 10 min, 15 min and 20 min were performed, and fluorescence value and corresponding H&E staining was used for evaluation. 10 min permeabilization time was chosen for the spatial gene expression experiment of mouse brain and E12.5 embryonic eye, respectively. No replicate was applied.
B. H&E staining of sections used for spatial expression.

Salus

A

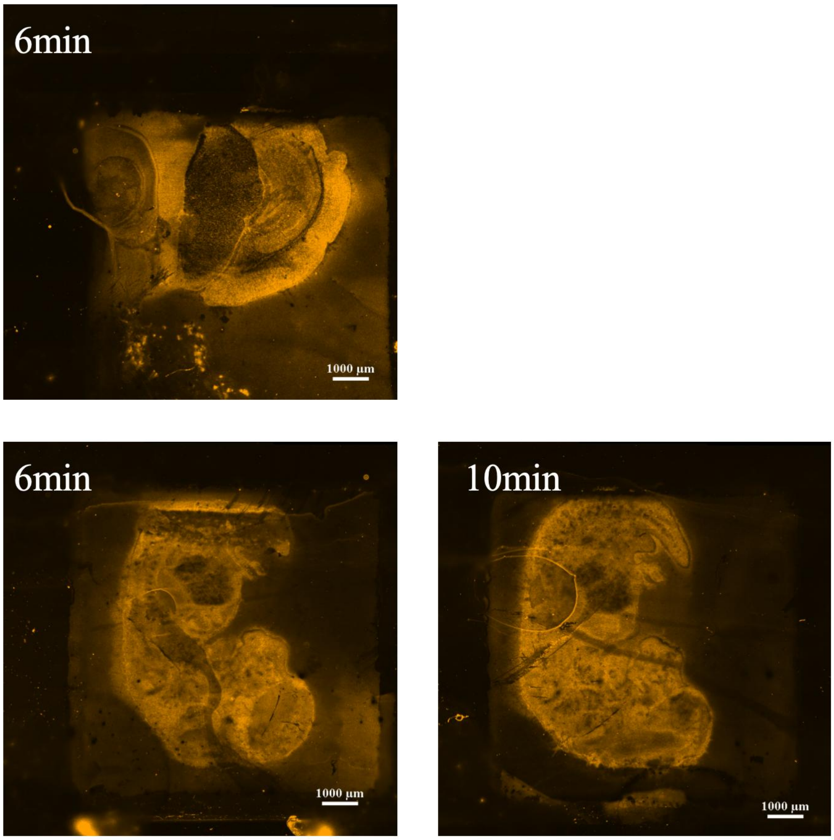

Supplementary Fig.5.

A. Tissue permeabilization were optimized before spatial gene expression experiment. Permeabilization for 6 min and10 min were performed, and fluorescence value and corresponding H&E staining was used for evaluation. 6 min permeabilization time was chosen for the spatial gene expression experiment of mouse brain and E12.5 embryonic eye, respectively. No replicate was applied.

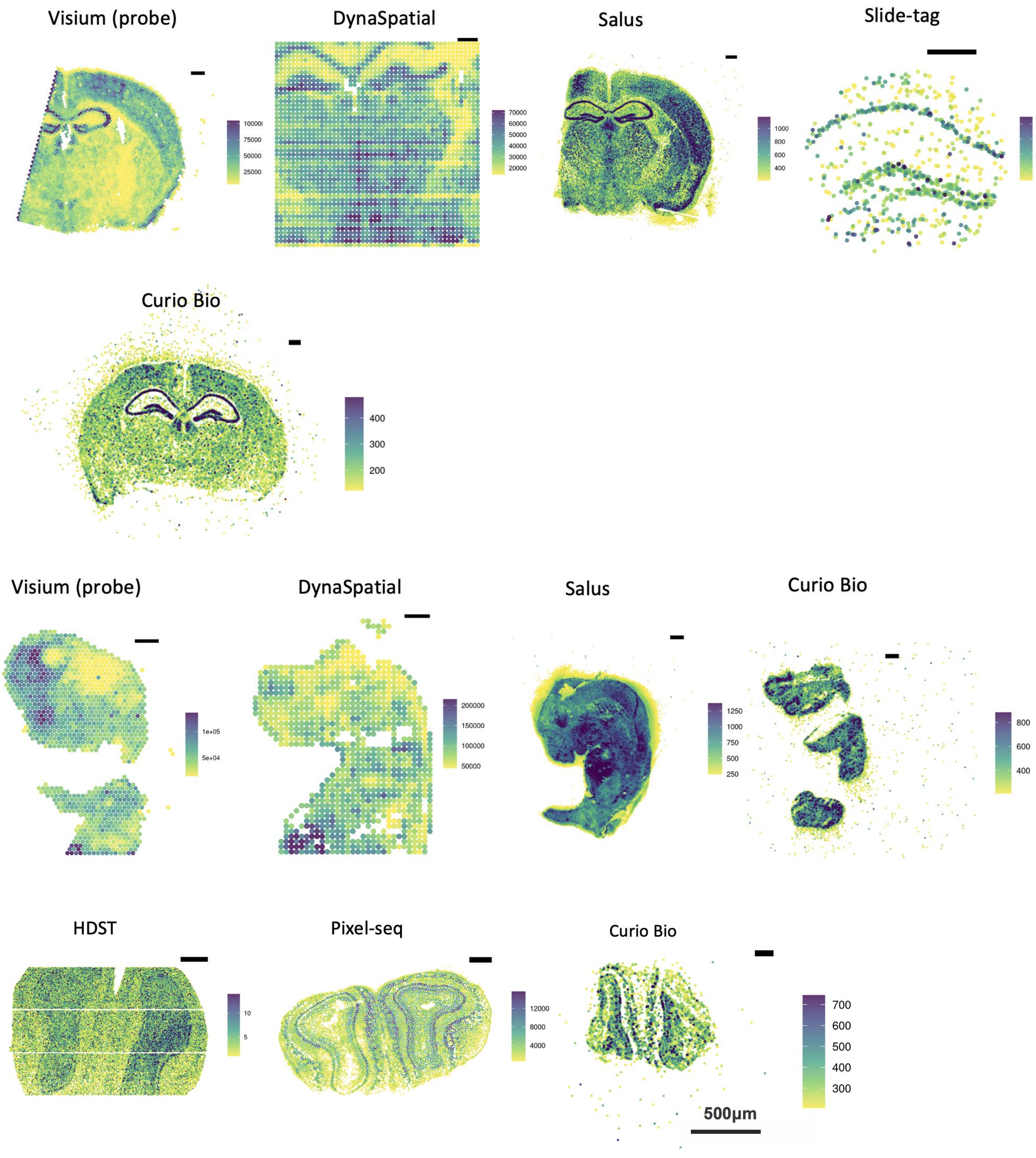

Supplementary Fig.6. The visualization of total counts across the spatial dimension for datasets generated using each platform for reference tissues is shown. The length of the black bar in the visualization corresponds to a distance of 500 microns.

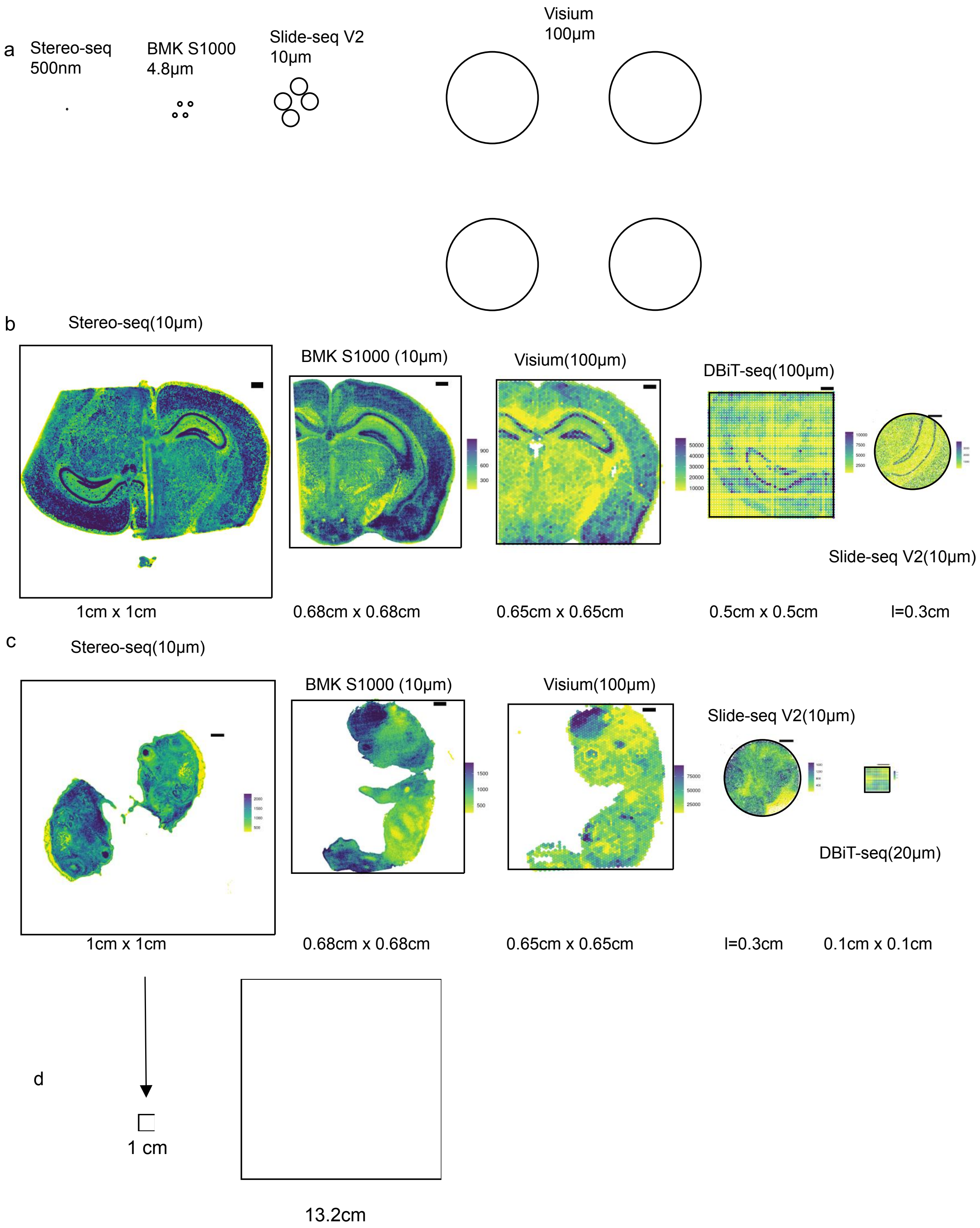

Supplementary Fig.7.

a) Spots are plotted according to their respective distances between spot centers and their positions on the chip.

The capture area for each sST method is depicted with a black box, overlaid with b) a brain sample and c) an embryo sample. The size of each box corresponds to the capture area information listed below, offering a comprehensive overview of the differences in capture areas.

d) The relatively visualization of the size between 1cm x 1cm chip compared to the 13.2cm x 13.2cm, which is the maximum size of the chip provided by Stereo-seq.

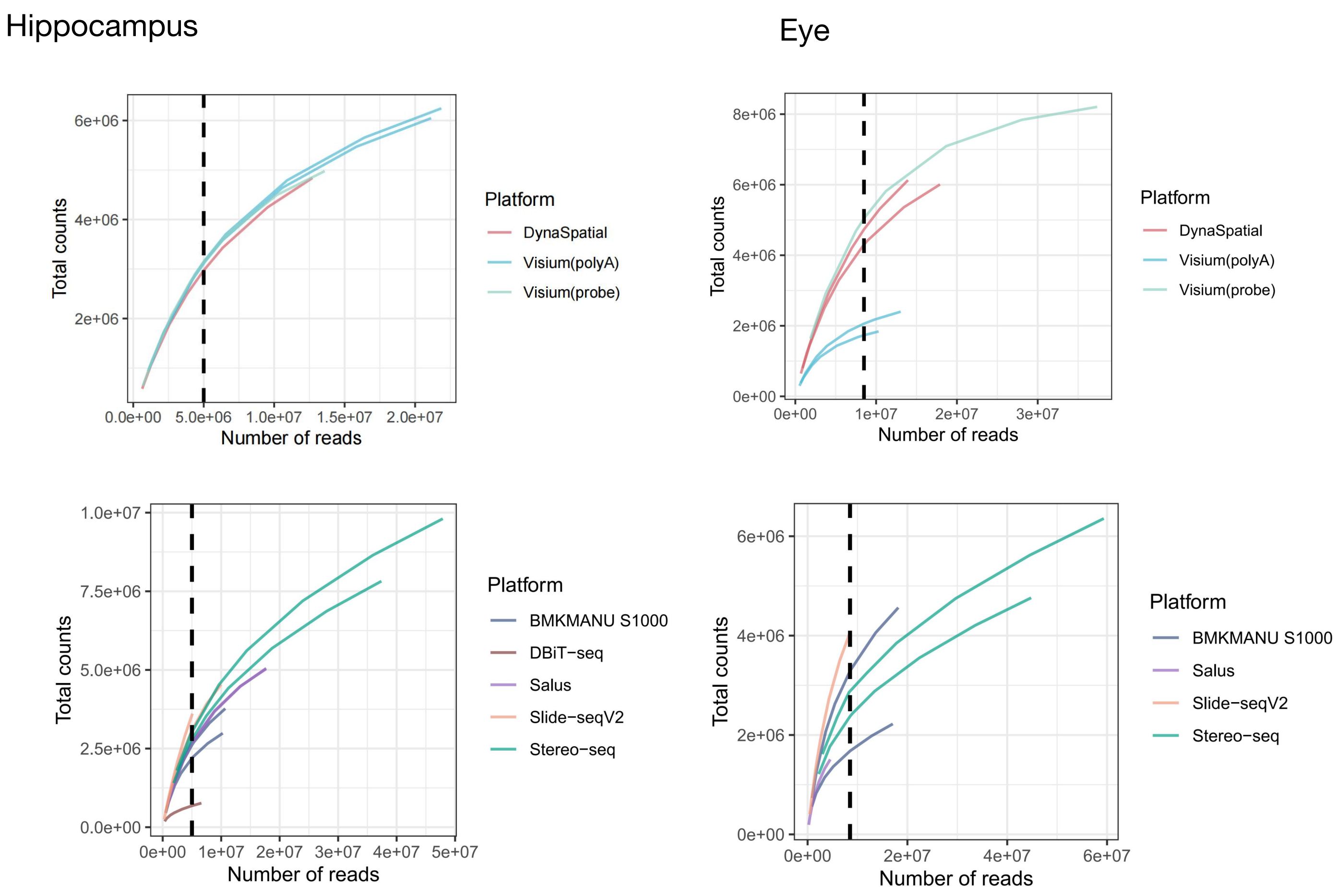

Supplementary Fig.8. Total UMI counts are presented as a function of stepwise downsampled sequencing depths for each platform. The data originates from mouse hippocampus and E12.5 mouse eye regions. A vertical dashed black line marks the read count used for generating the subsequent downsampled data. Methods that achieve 20 micron-resolution and those did not achieve are plotted separately.

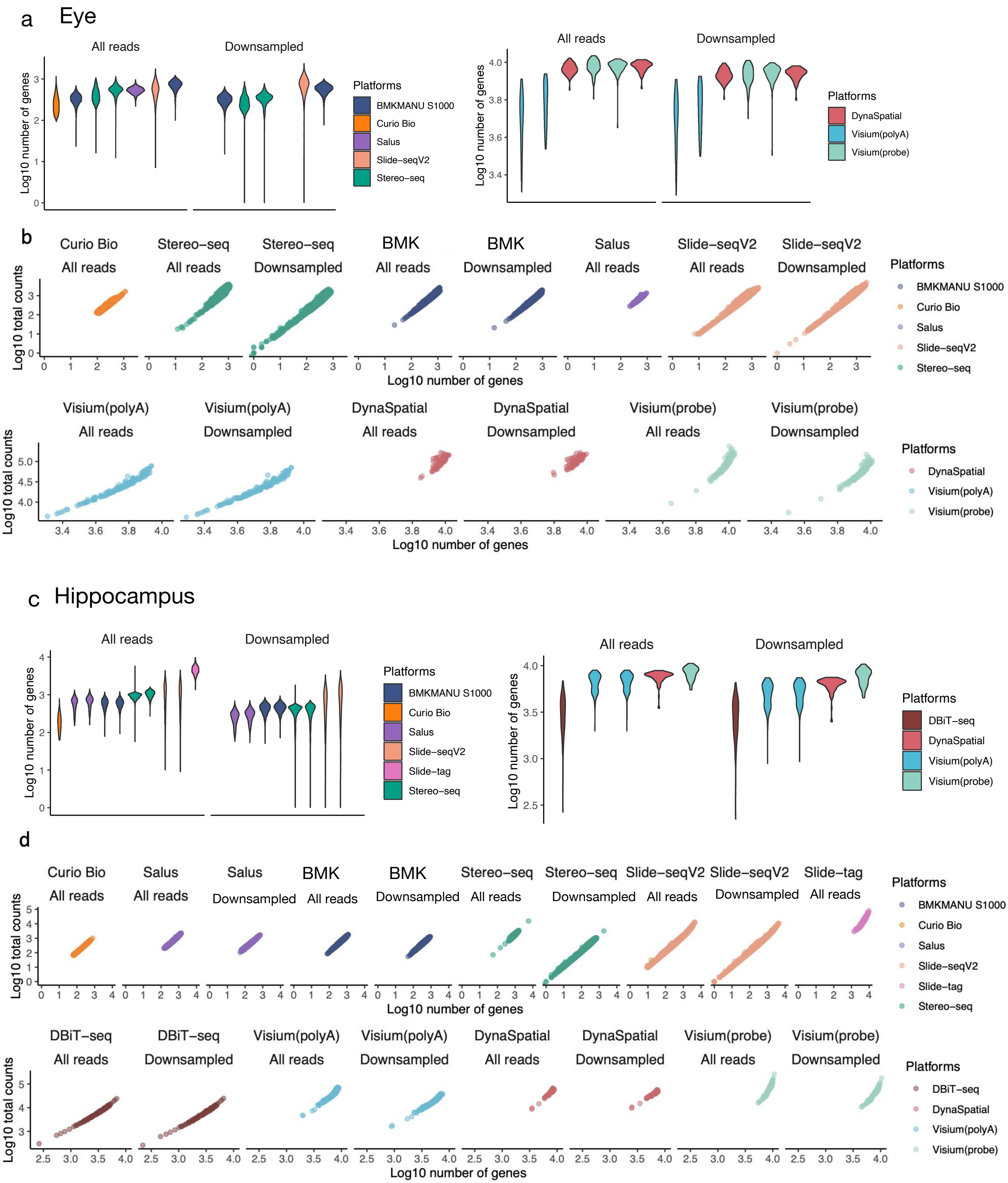

Supplementary Fig.9. a) In a selected region of the E12.5 mice eye, the left panel shows the log10-transformed number of detected genes per spot, using data from all reads. In the right panel, the same information is presented using downsampled data. Notably, the resolution for methods in the left panel is 10μm, while for other platforms in the right panel, it is 100μm.

The relationship between the log10-transformed number of genes and log10-transformed total counts per spot is individually plotted in b).

c) For a selected region in the adult mice hippocampus, corresponding plots are provided in c) and d). The same format and spot size considerations apply.

a

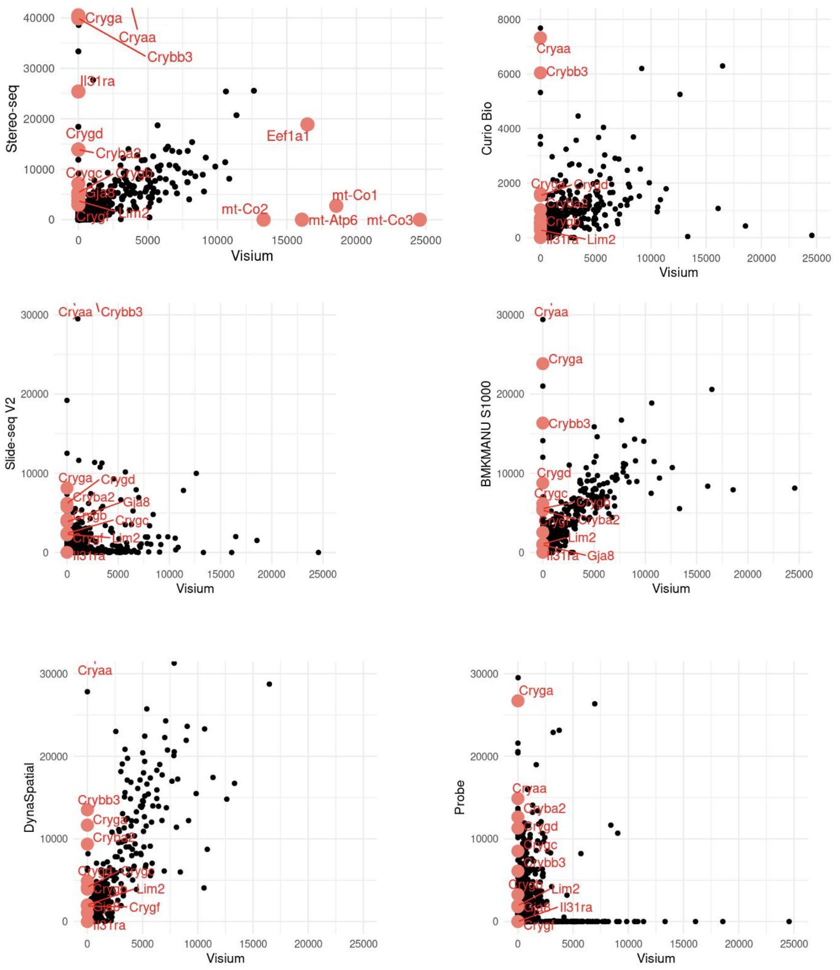

b *scPipe* processed Visium(polyA) data

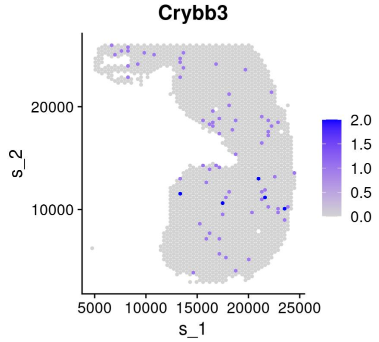

Supplementary Fig.10. a) Expression levels of all detected genes are compared between Visium(polyA) and other platforms. Each dot represents a gene, shown in black. Genes that display expression at the 90th percentile in other platforms but are at the 10th percentile in Visium(polyA) are highlighted in red and labeled with their gene symbols.
b) Expression of the example gene Crybb3, which exhibited bias as illustrated in plot a), is visualized in spatial reduction. Preprocessing was performed using *scPipe* instead of *SpaceRanger*. Notably, unexpected expression of Crybb3 was observed in Visium(polyA) data, contrasting with the expected high expression in the lens, consistently observed in data generated by other platforms.

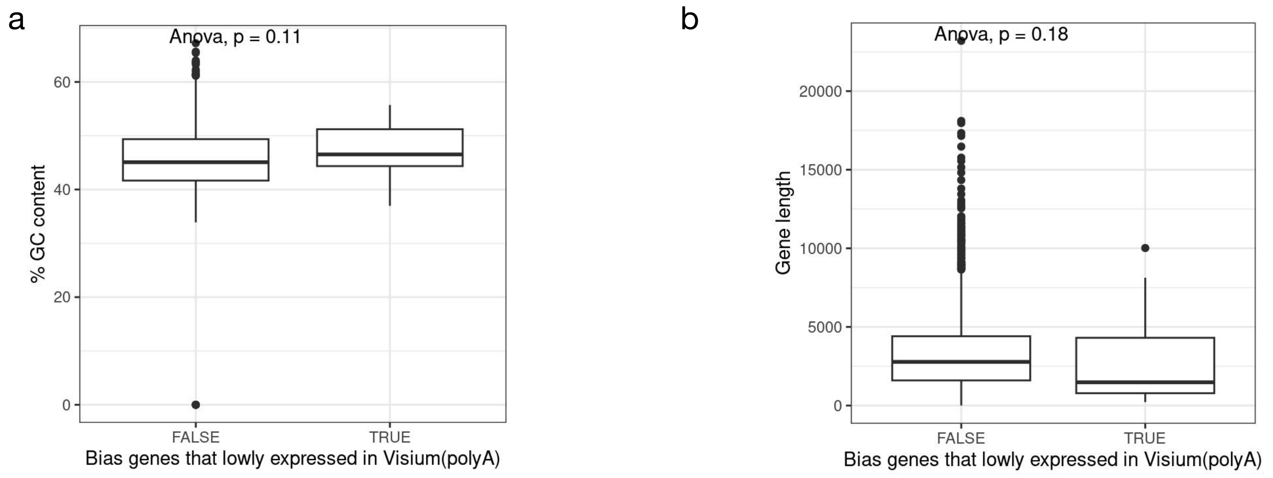

Supplementary Fig.11. In the initial step of our analysis, we identified genes that exhibited expression across all platforms, surpassing the 90th percentile threshold in terms of total counts. Within this carefully curated gene set, we further classified them based on their expression levels specifically within the Visium(polyA) dataset. Genes with expression levels below 30 were categorized as bias genes, showing lower expression specifically within the Visium(polyA) dataset, while the remaining genes formed the basis for our comparative analysis. Subsequently, we conducted two-sided ANOVA analyses to explore the relationships between the GC content percentage, gene length and the selected genes, using the 90th percentile threshold. The box denotes the interquartile range (IQR), the range between the 25th percentile and 75th percentile, with the median value; whiskers indicate the maximum and minimum value within 1.5 times the IQR. Outliers are denoted by black circles. N=37 for selected, and N=3047 for not selected genes.

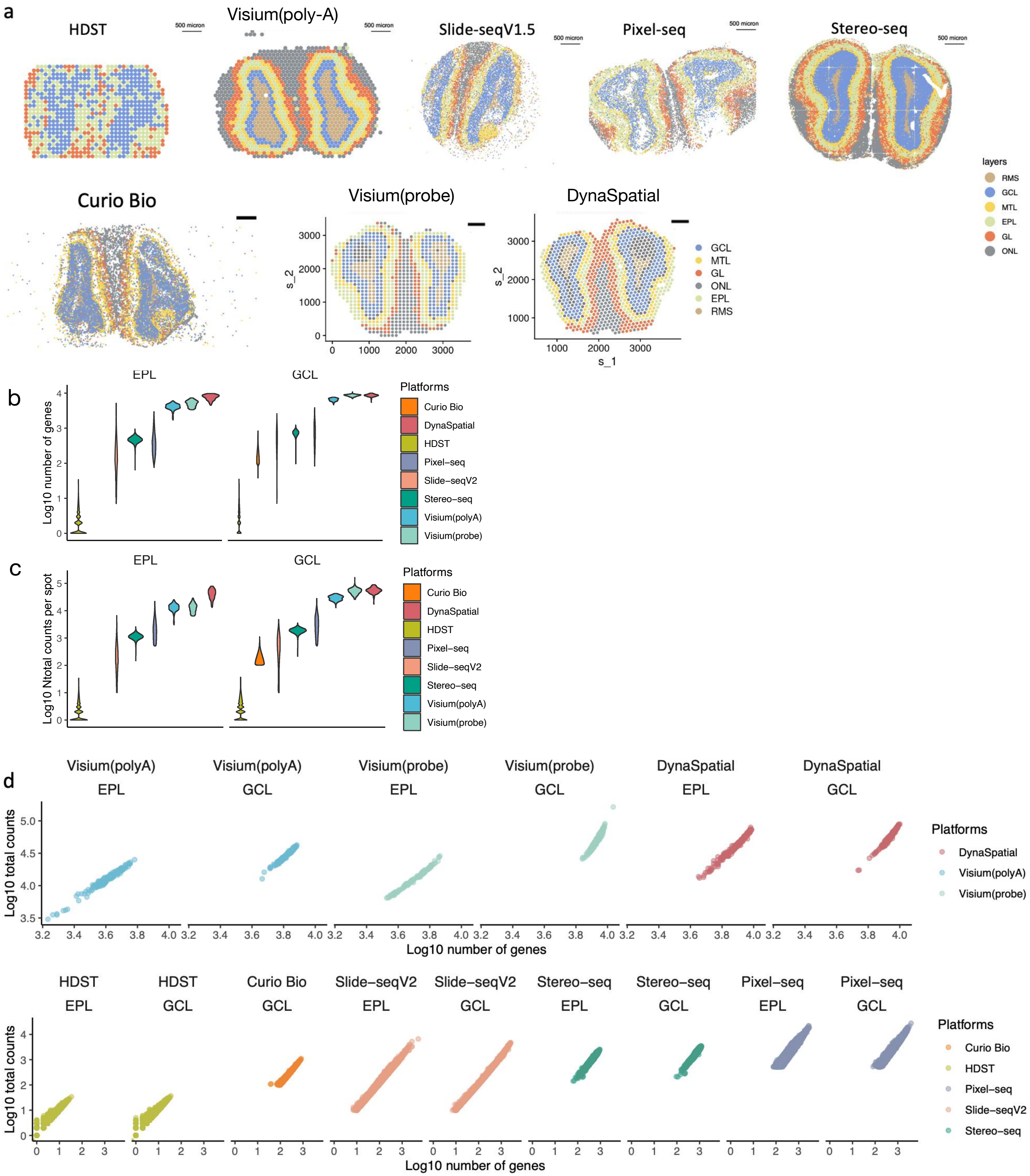

Supplementary Fig.12. Data obtained from various platforms for the mouse olfactory bulb were processed and annotated. The spatial reduction plots, color-coded by annotated layers, are presented individually for each platform in a).

In b), the log10-transformed number of detected genes per spot, calculated from the data of all reads used, is depicted separately for the External Plexiform Layer (EPL) and the Granule Cell Layer (GCL). These layers exhibit different gene density and count patterns.

c) Log10-transformed total counts per spot, derived from the data of all reads used, are showed separately for EPL and GCL in c). Notably, the spot size for Visium, DynaSpatial is 50μm x 50μm, whereas for other platforms, it is 10μm x 10μm.

d) The relationship between the log10-transformed number of genes and the log10-transformed total counts per spot is individually illustrated in b).

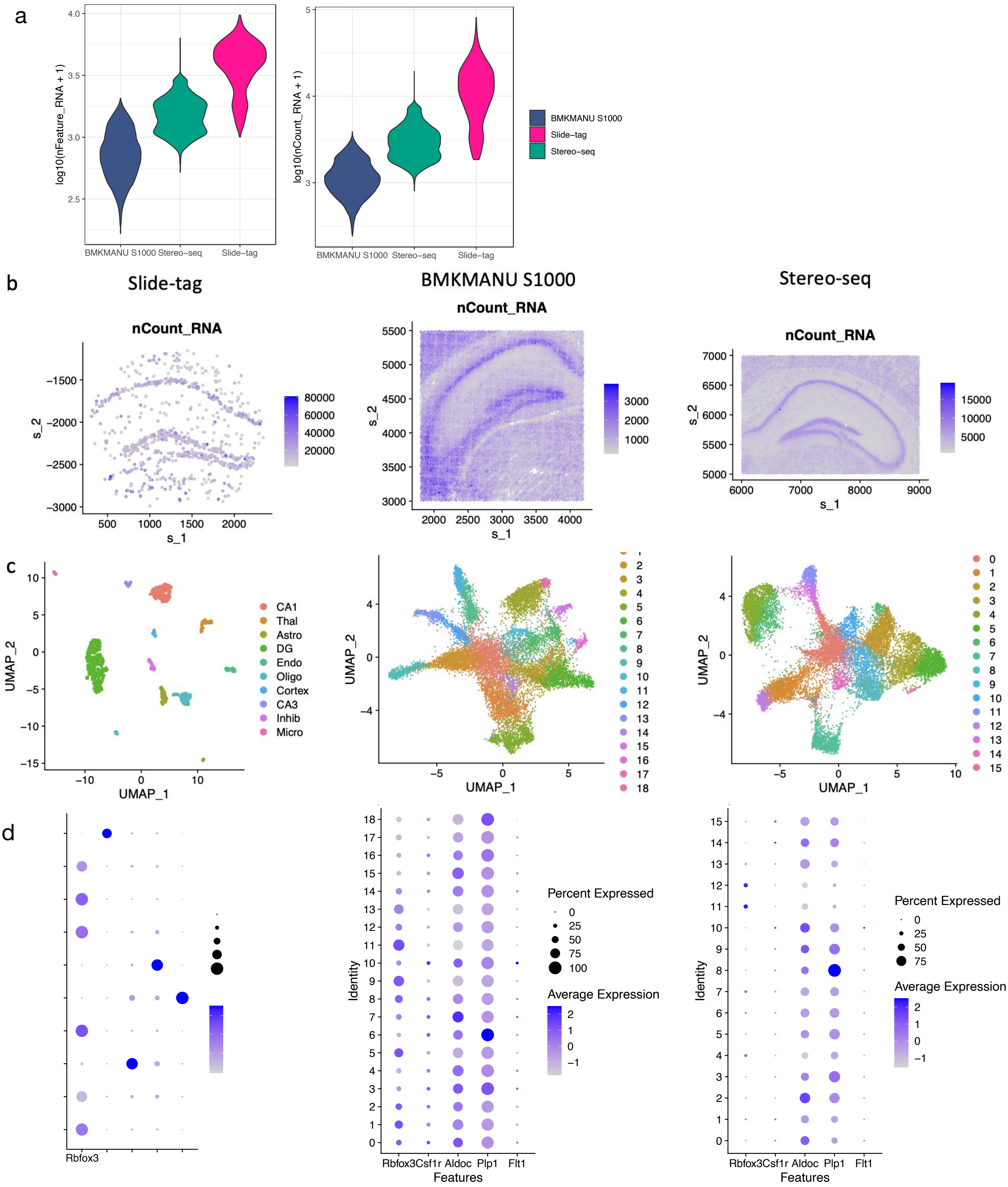

Supplementary Fig.13. In panel a), spots from BMK S1000 and Stereo-seq have been aggregated and binned to a 20-micron resolution. They are presented alongside data from slide-tag in violin plots, showcasing log-transformed counts of features and total counts per unit. b) provides a visual representation of the spatial distribution of captured spots within the tissue sample.

In c), UMAP plots display clustering results generated from each of these datasets.

In d), the expression levels of selected marker genes specific to different cell types within the cortex are depicted separately for each cluster.

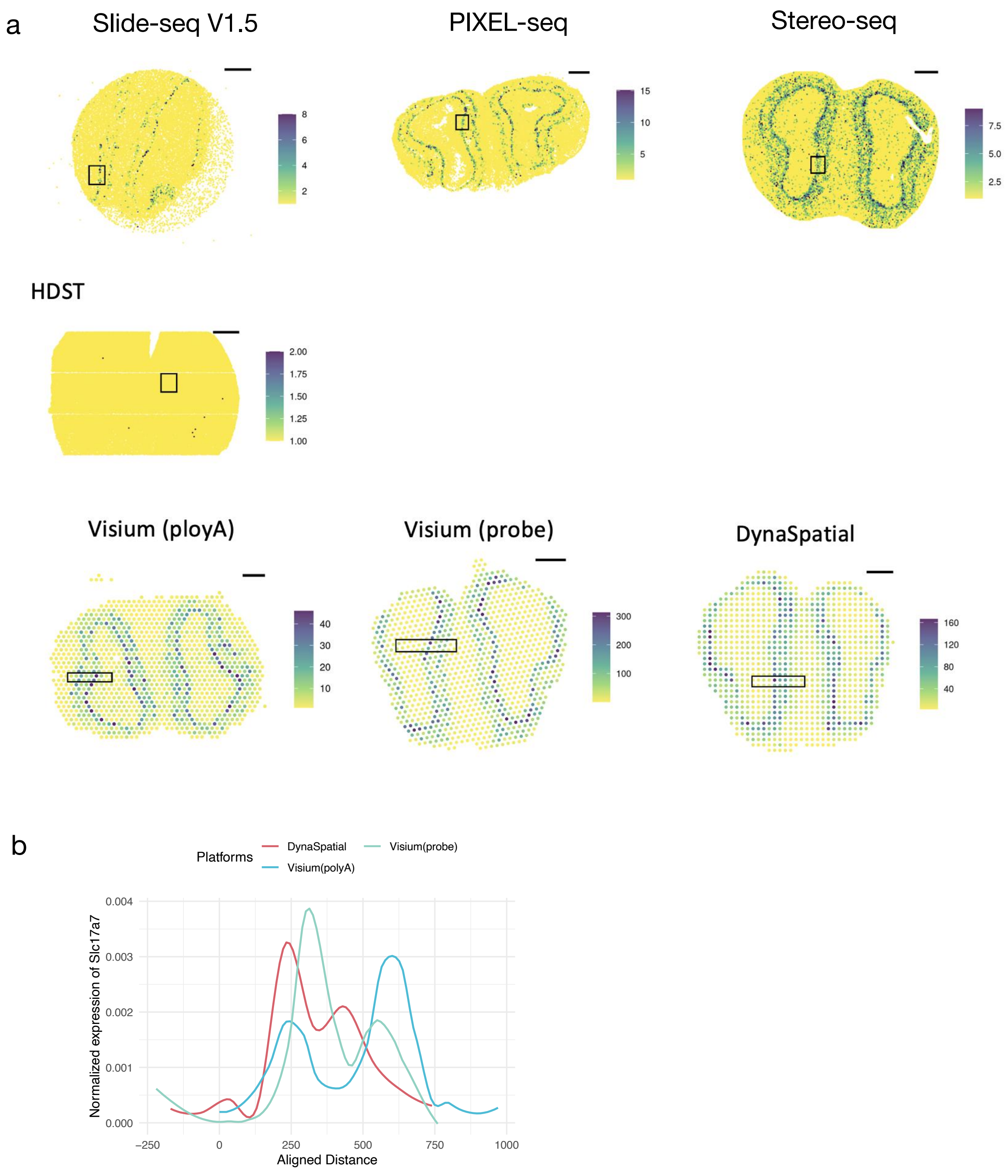

Supplementary Fig.14. a) Spatial reduction plots depict the expression of *Slc17a7* across different data platforms, with color coding reflecting raw count values. Black boxes delineate specific regions chosen for diffusion calculations. b) Expression of *Slc17a7* across selected modality is illustrated in a density plot. Comparisons are made between results obtained using data from methods as shown in the bottom panel of a).

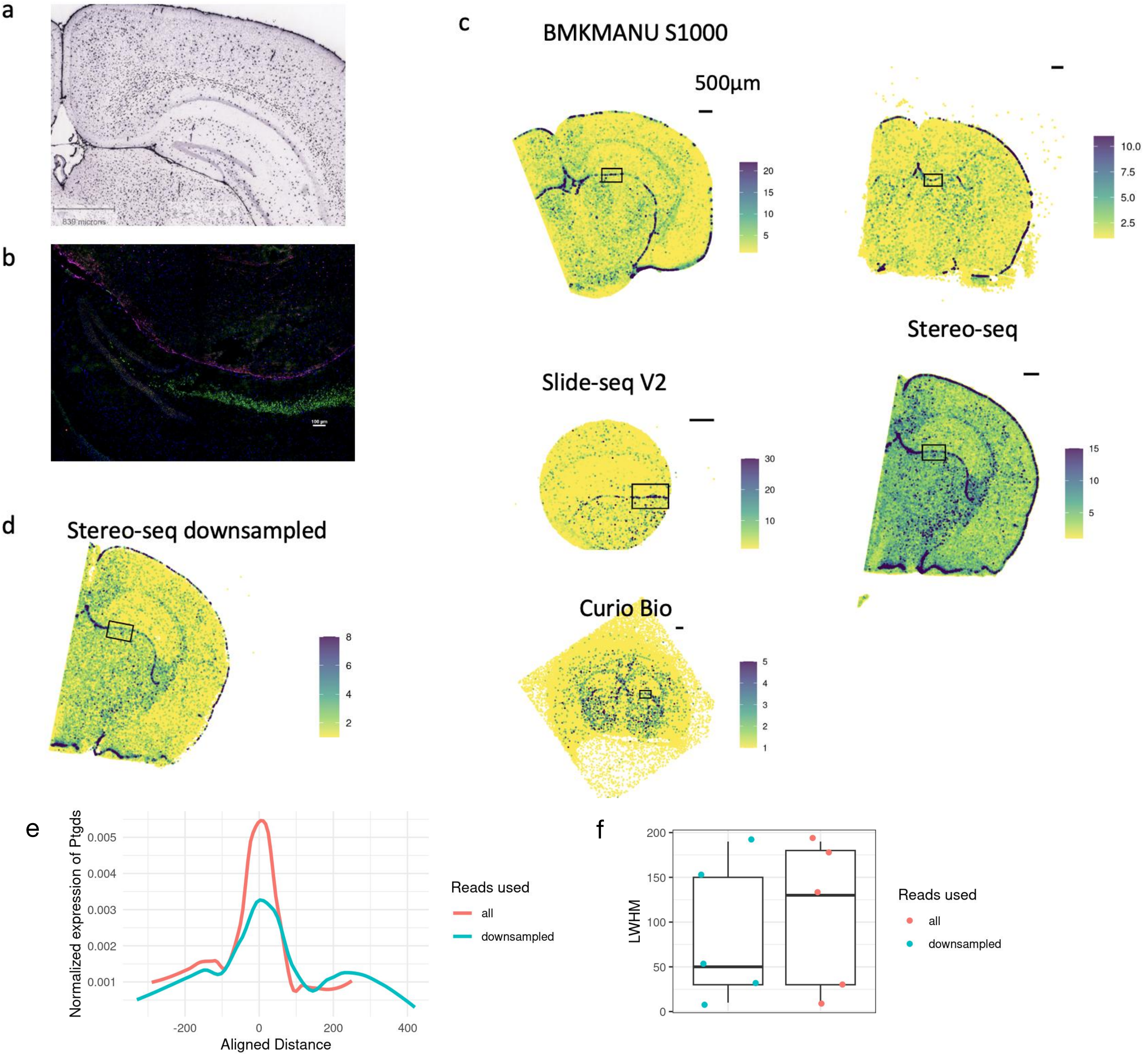

Supplementary Fig.15. a) ISH image showing expression of *Ptgds* from https://mouse.brain-map.org/experiment/show/79567709.
b) ISH image with expression *Ptgds* shown in purple, *Prox1* shown in red, *Slc17a7* shown in green and nuclei shown with DAPI. No replicate was applied.
c) Spatial reduction plots depict the expression of *Ptgds* across different data platforms, with color coding reflecting raw count values. Black boxes delineate specific regions chosen for diffusion calculations.
d) Spatial reduction plots display the expression of *Ptgds* on downsampled data from stereo-seq, generated using reads comprising only 14% of the total number of reads.
e) The average expression of *Ptgds* across selected modalities is illustrated in a density plot. Comparisons are made between results obtained using all reads and downsampled reads.
f) LWHM values were computed for the same sets of modalities and are presented in boxplots, where each dot represents a modality (n=5). The box denotes the interquartile range (IQR), the range between the 25th percentile and 75th percentile, with the median value; whiskers indicate the maximum and minimum value within 1.5 times the IQR.

a

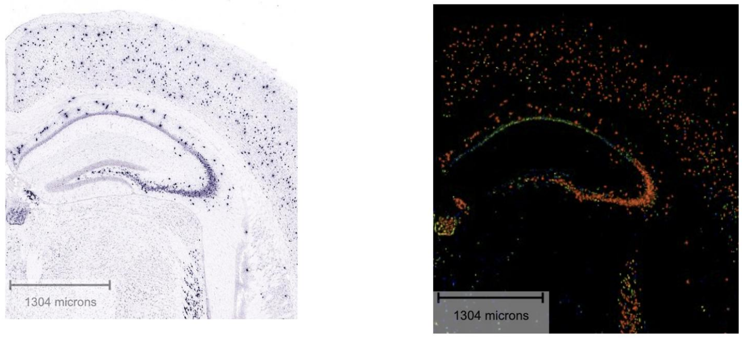
1304 microns
1304 microns

b

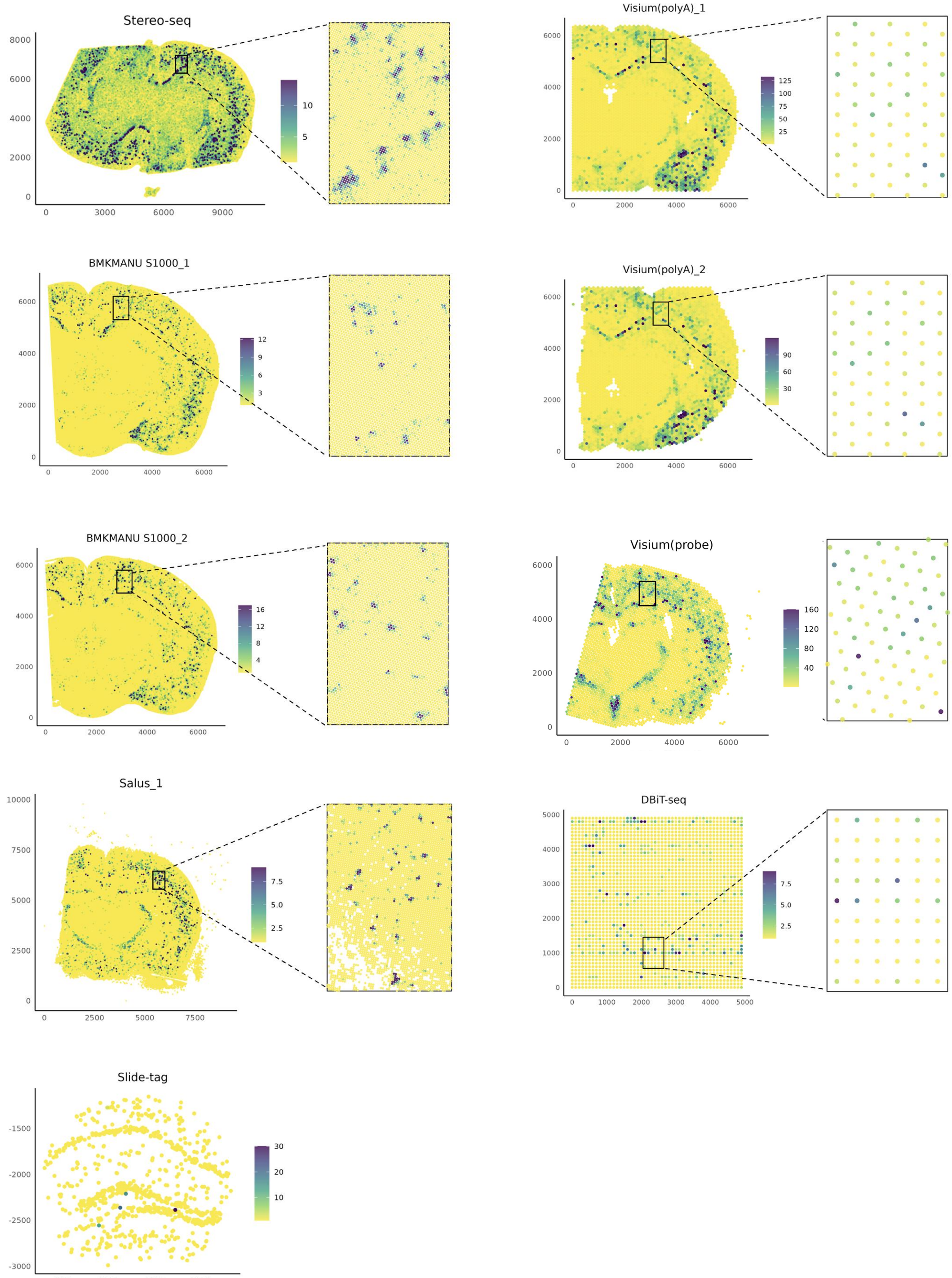
Stereo-seq
Visium(polyA)_1
BMKMANU S1000_1
Visium(polyA)_2
BMKMANU S1000_2
Visium(probe)
Salus_1
DBiT-seq
Slide-tag

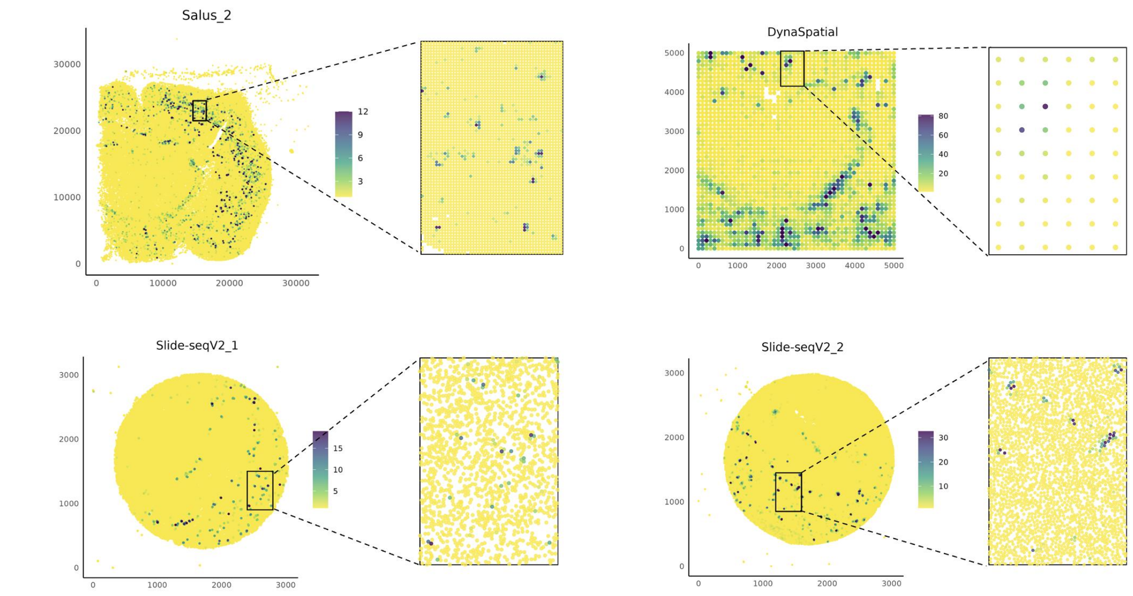

c

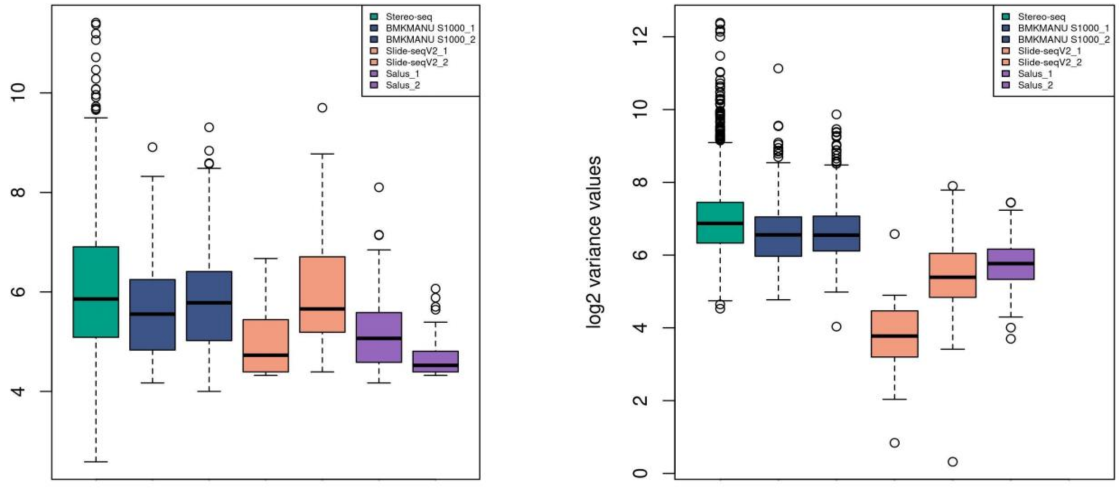

Supplementary Fig.16. a) presents the expression profiles of *Sst* in the mouse brain through ISH staining. These images are sourced from the Allen Brain Cell Atlas (https://portal.brain-map.org/).

b) provides a comprehensive depiction of *Sst* expression across all our samples. For a closer examination of *Sst* expression patterns, specific plots have been zoomed in, allowing for a more detailed visualization.

c) We calculated the average UMI counts within clusters expressing *Sst*, which are indicative of potential Sst neurons. To provide further insights into the diffusion of expression, we also calculated the variance in the spatial distribution of UMI counts within these clusters. The box denotes the interquartile range (IQR), the range between the 25th percentile and 75th percentile, with the median value; whiskers indicate the maximum and minimum value within 1.5 times the IQR. Outliers are denoted by black circles.

For each sample, the number of clusters used here are n= 1021, 283, 331, 24, 68, 154, 83 accordingly.

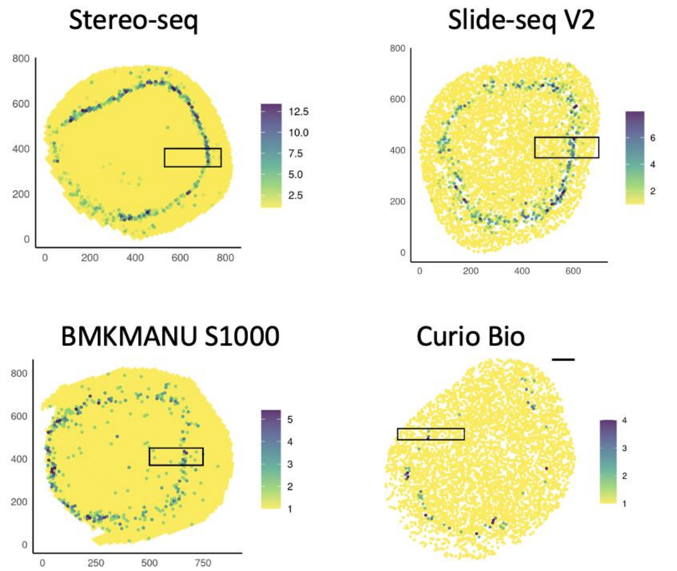

Supplementary Fig.17. Spatial reduction plots illustrate the expression of *Pmel* across diverse platforms, with colors representing raw count values in mice embryo eyes. Black boxes highlight specific regions chosen for diffusion calculations.

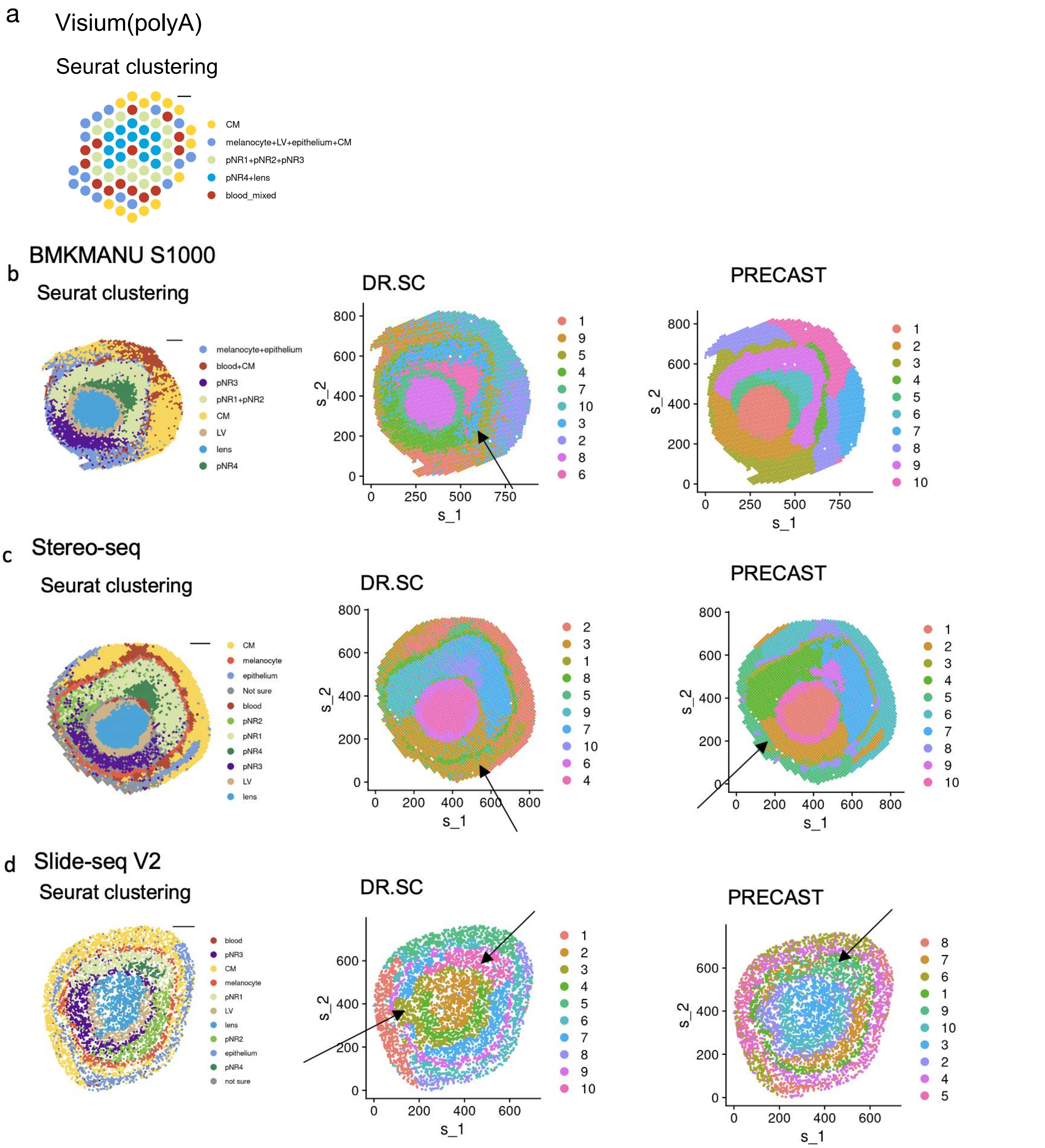

Supplementary Fig.18. Count matrices from individual platforms underwent quality control and normalization procedures. Subsequently, clustering was performed using three selected methods. Dimplot visualizations with spatial reduction, colored by clustering results, are presented from left to right, corresponding to Seurat clustering, DR.SC, and PRECAST for the following samples: a) 10X Visium b) BMKMANU S1000 c) Stereo-seq d) Slide-seq V2.

Within each panel, the arrow bar indicates differences between clustering results.

Notable findings include:

For BMK data, DR.SC successfully identified a cluster of melanocytes, whereas the other two methods failed to do so.

In the case of stereo-seq data, DR.SC combined pNR3 and epithelium cells and grouped spots with unknown annotation together. PRECAST missed a set of melanocytes located near the region enriched with spots for which annotation remains uncertain.

In Slide-seq V2 data, both DR.SC and PRECAST failed to detect pNR4.

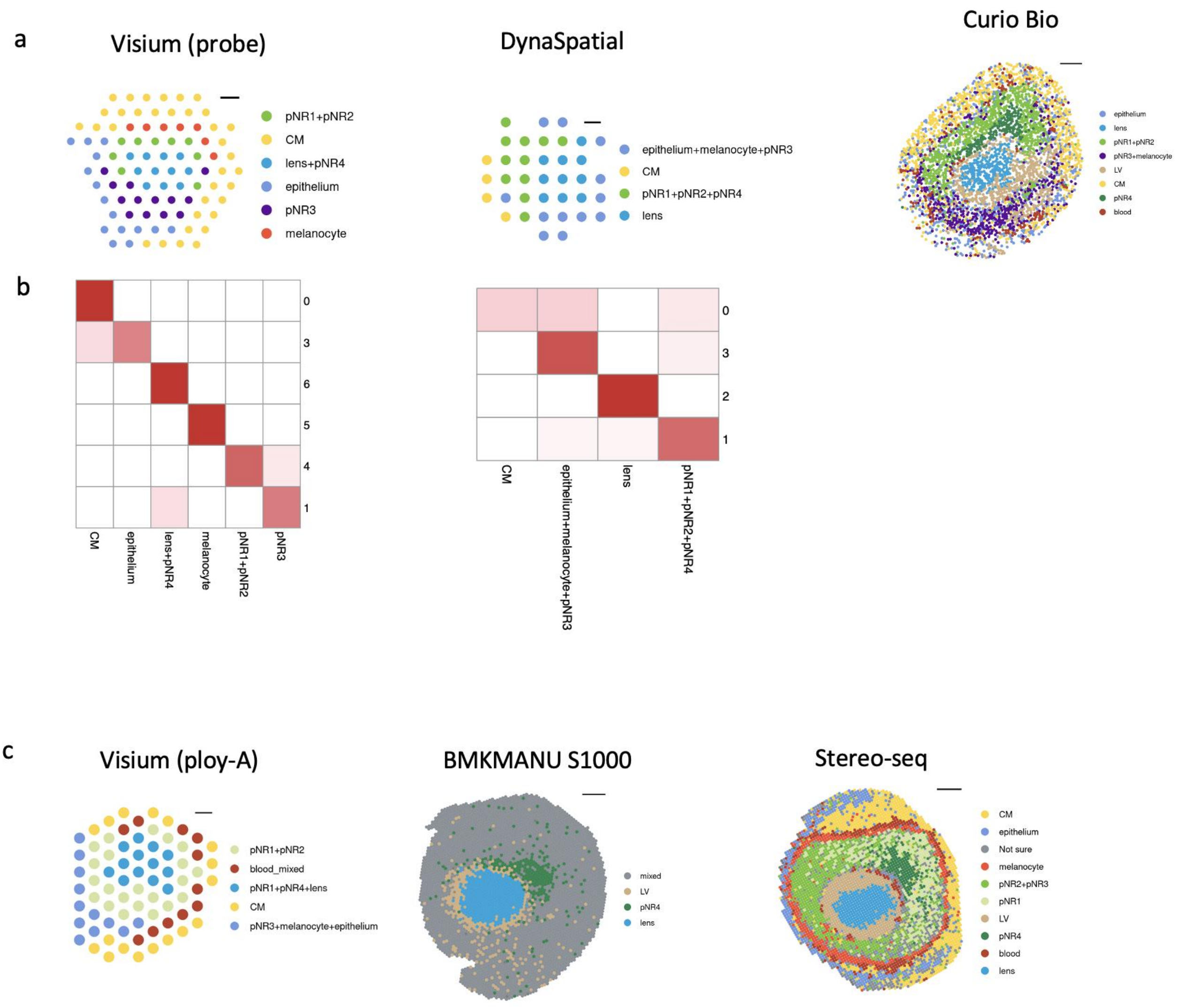

Supplementary Fig.19. a) and c) Expression profiles generated from different platforms have been processed and annotated. Clustering results are presented in their spatial reduction for samples that are not featured in the main plot.
b) For the two methods on the left side of panel a), clustering was carried out on downsampled eye data from each platform, ensuring an equal total read count across platforms in the eye area. The correspondence between annotations derived from clustering based on all reads and clustering based on downsampled data is visually represented in a heatmap. The count of spots involved in this correspondence is displayed after log10 transformation, without any additional scaling applied.

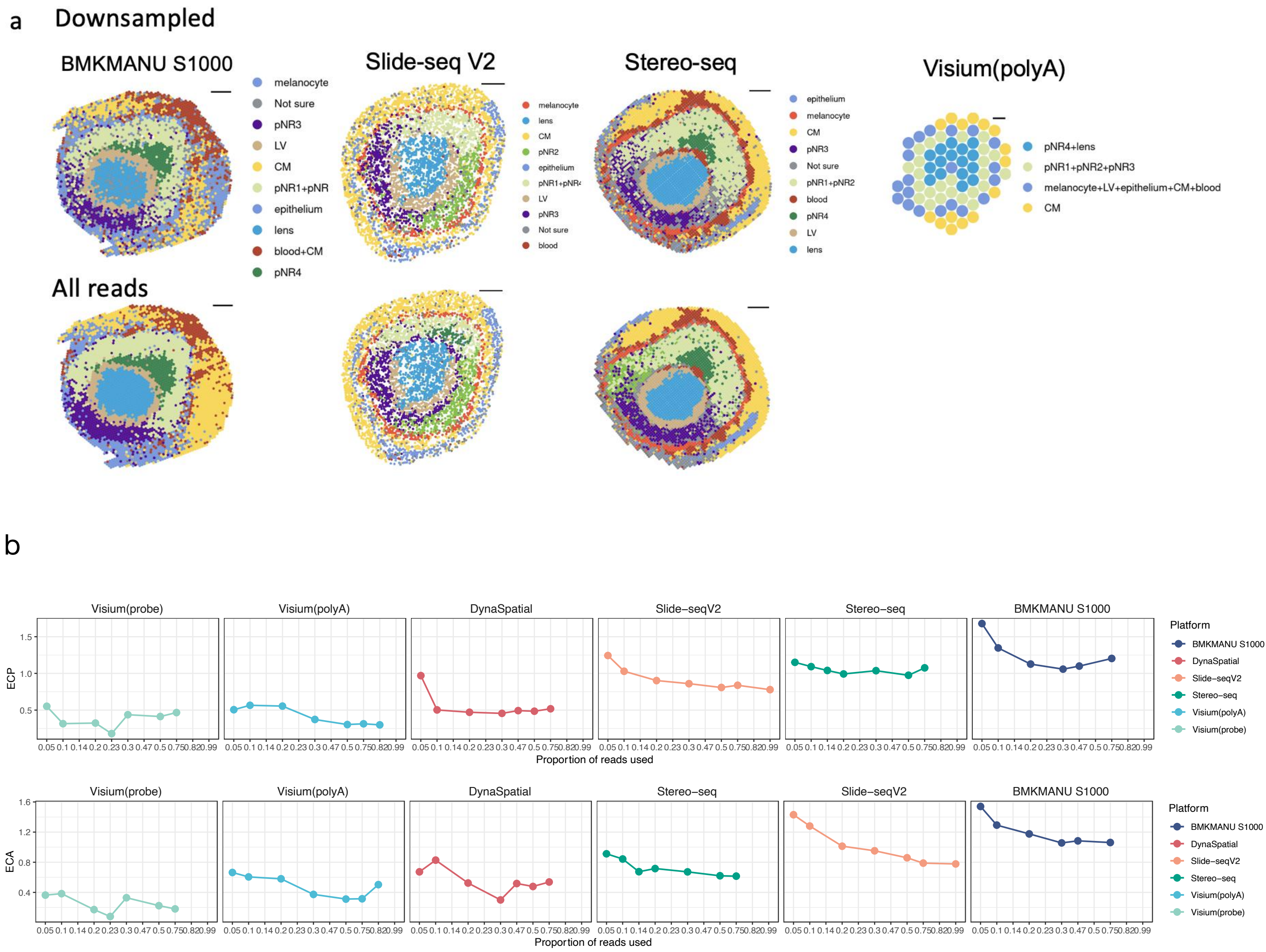

Supplementary Fig.20. Downsampled samples, corresponding to those depicted in Figure 3, were utilized for subsequent analyses. Seurat clustering was applied to these downsampled samples, and the clustering results are presented in spatial reduction plots in a), with spots color-coded by annotated cell states. Corresponding spatial reduction plots are displayed underneath.

In general, the major cell states are still discernible in downsampled datasets, reflecting the robustness of the analysis.

To assess clustering consistency between downsampled and full-read datasets, b) ECP and ECAscores were calculated and plotted, with each dot representing a downsampled dataset. Lower ECA and ECP scores indicate more consistent results.

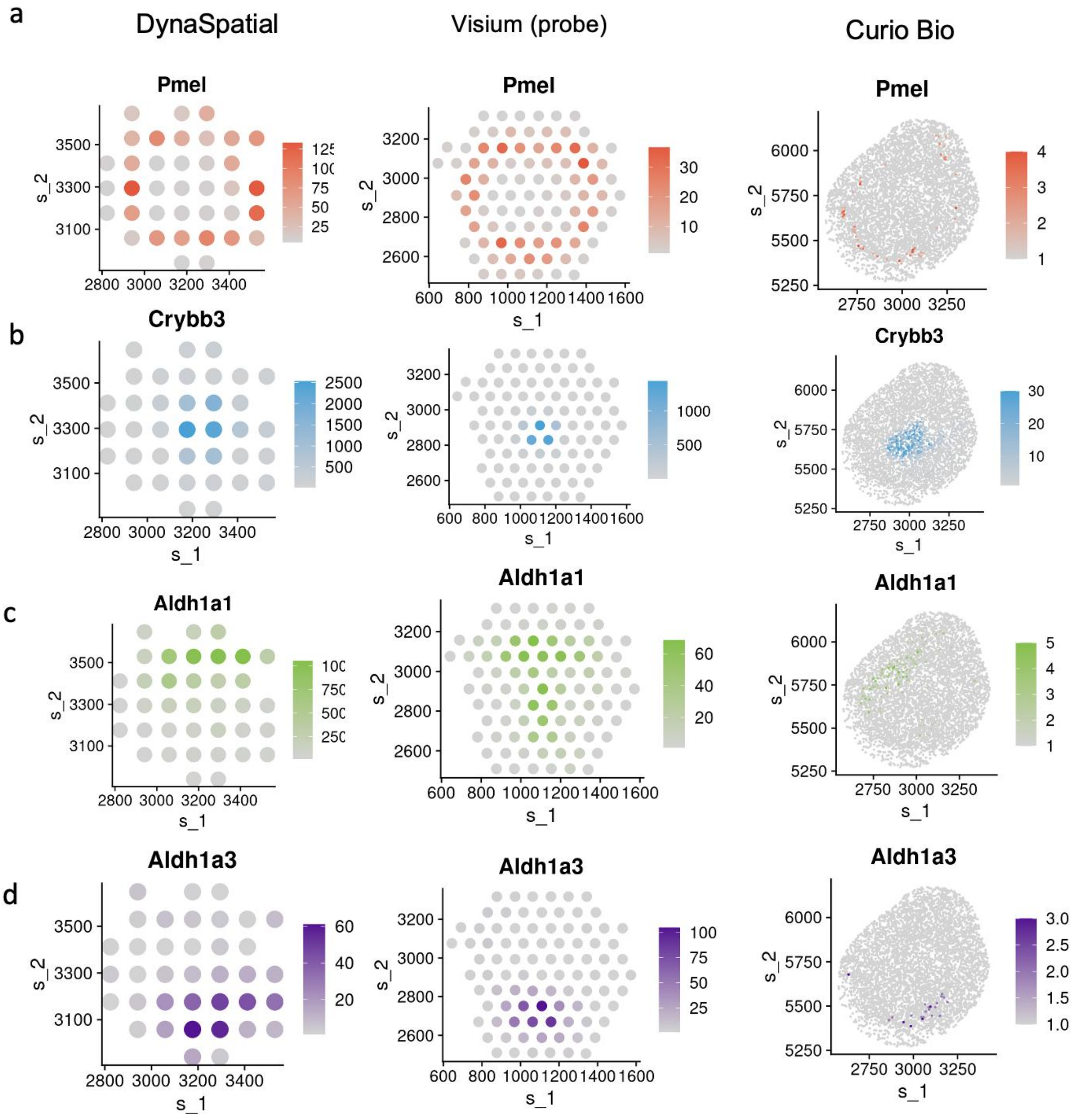

Supplementary Fig.22. Expression of *Pmel* a) , *Crybb3* b), *Aldh1a1* c) and *Aldh1a3* d) were plotted in their spatial reduction for data generated from different platforms and the color denotes the values of raw counts of selected genes.

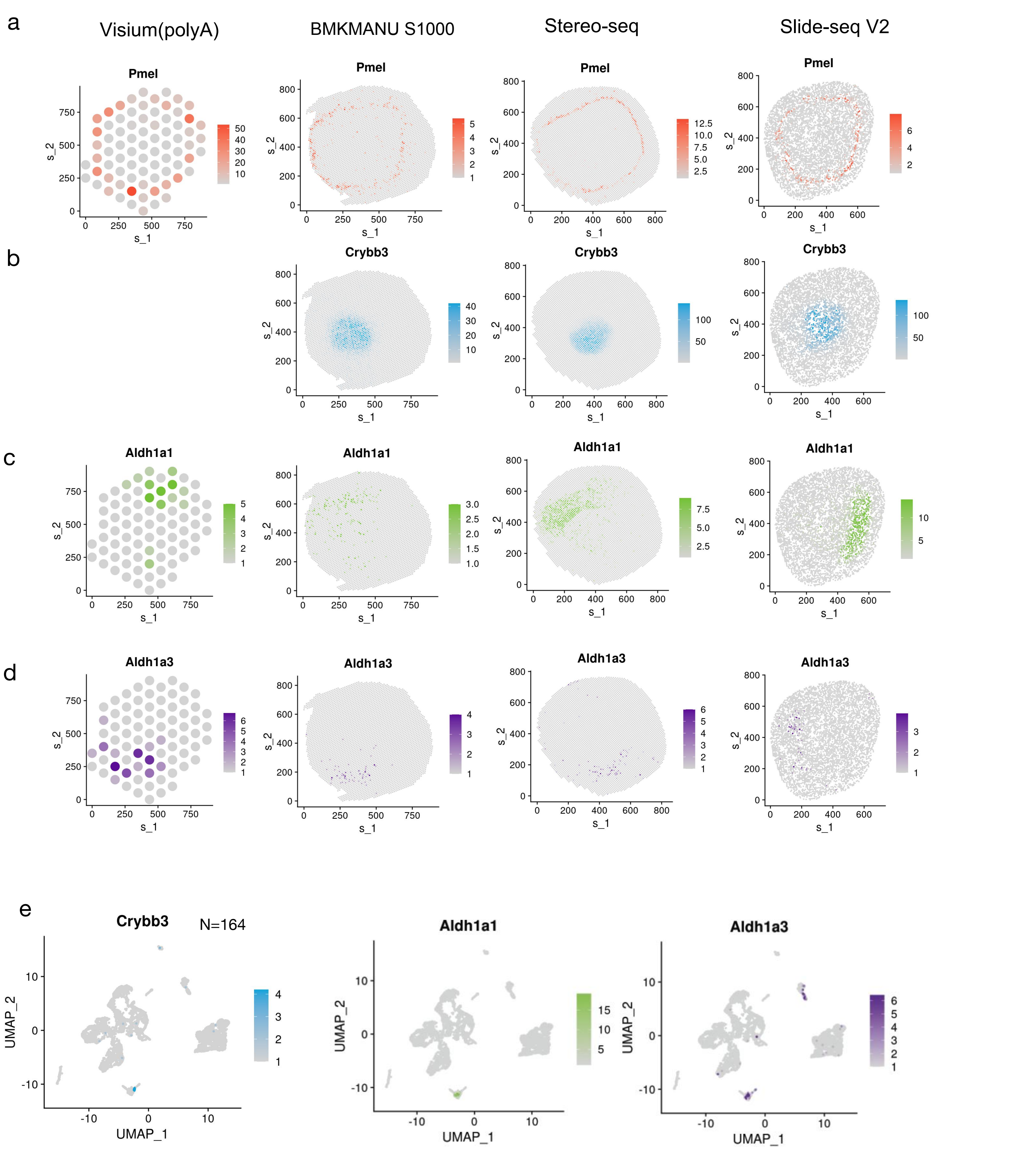

Supplementary Figure 21. Expression of *Pmel* a) , *Crybb3* b), *Aldh1a1* c) and *Aldh1a3* d) were plotted in their spatial reduction for data generated from different platforms and the color denotes the values of raw counts of selected genes.

With Stereo-seq data as an example, spatial expression profiles of *Aldh1a1* and *Aldh1a3*, which are expressed in pNR2 and pNR3 are shown at 10 μm resolution. The number of spots with the expression of *Aldh1a1* above 0 is 1,329, and of *Aldh1a3* above 0 is 217.

e) Expression plot *Crybb3, Aldh1a1. Aldh1a3* were also plotted in UMAP for snRNA-seq data and the number of cells with expression of *Crybb3* above 0 is labelled on the right topin the left panel. The number of cells having an expression of *Aldh1a1* above 0 is 93, and of *Aldh1a3* above 0 is 161.

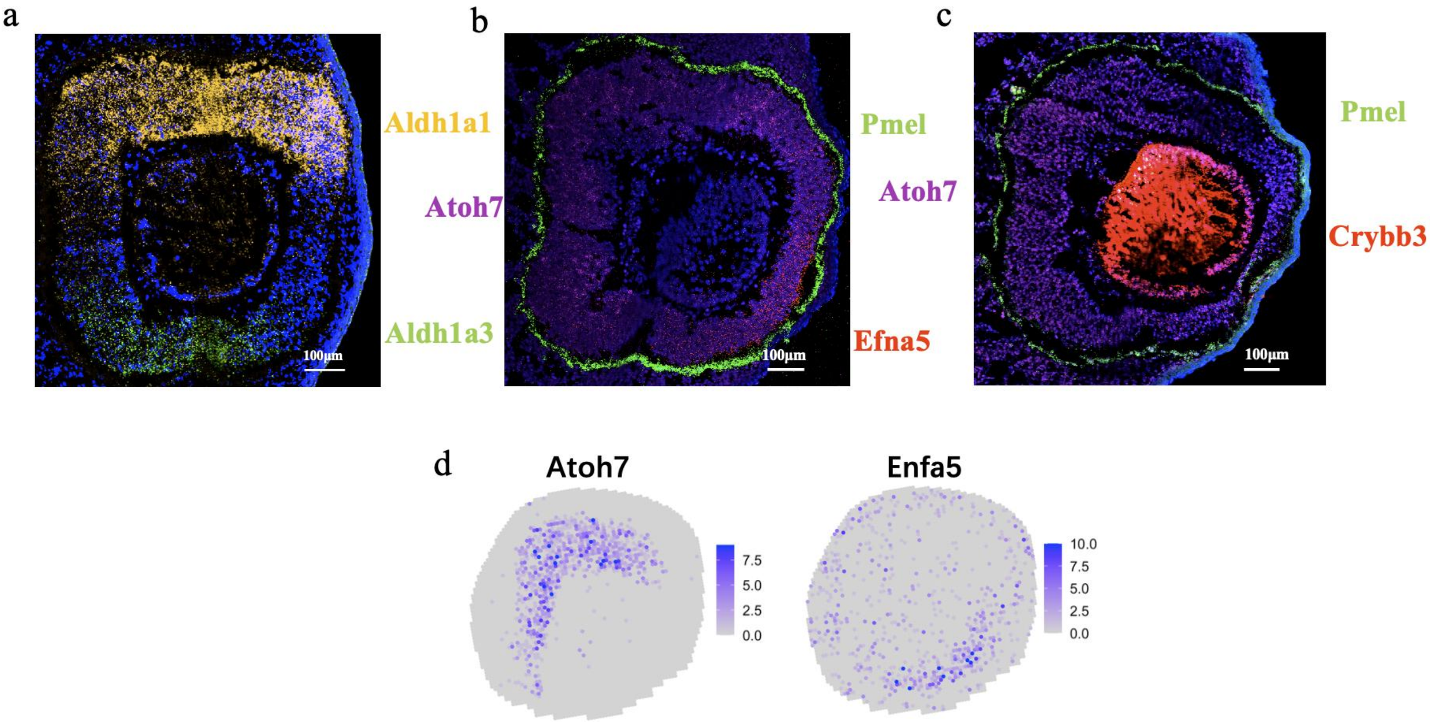

Supplementary Fig.23. In situ imaging of E12.5 mouse embryo eye.(a) The orange-red color represents the marker gene Aldh1a1,green represents the marker gene Aldh1a3, blue represents the nucleus. Scale bar, 100μm. (b) The red color represents the marker gene Efna5, green represents the marker gene Pmel, purple represents the marker gene Atoh7, blue represents the nucleus. Scale bar, 100μm. (c) The red color represents the marker gene Crybb3, green represents the marker gene Pmel, purple represents the marker gene Atoh7, blue represents the nucleus. Scale bar, 100μm. (d) Spatial expression of Atoh7 and Enfa5 in Stereo-seq data. No replicate was applied.

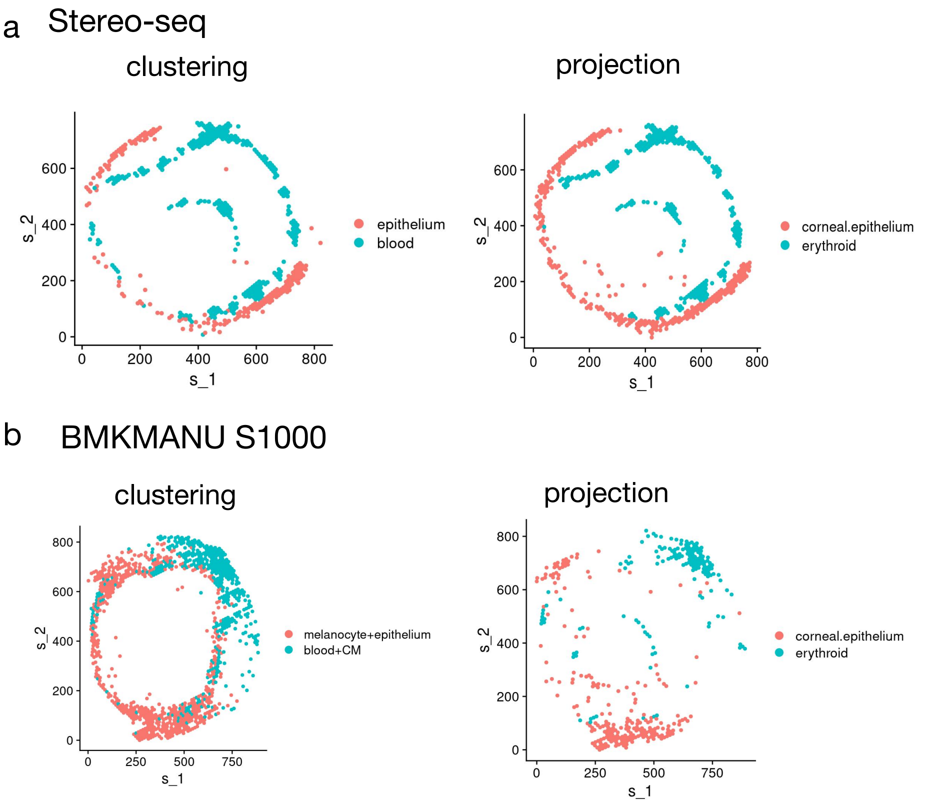

Supplementary Fig.24.
Clustering results generated by Seurat related to epithelium and blood and projection results based on snRNA-seq were plotted from left to right for data of a) Stereo-seq, b) BMKMANU S1000 accordingly.

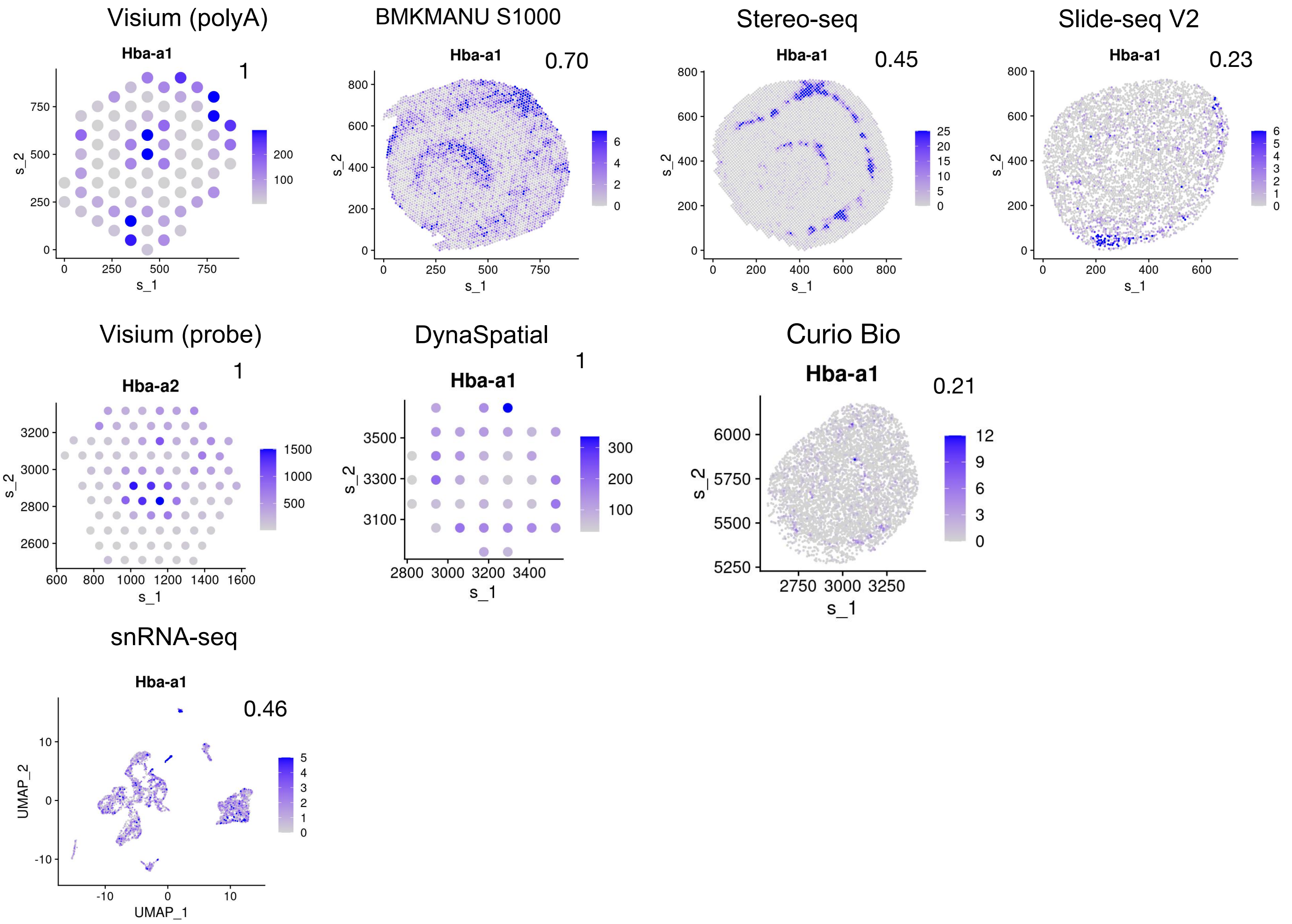

Supplementary Fig.25. Expression of Hba-a1 in data generated from different plotforms were plotted in their spatial reduction. The color denotes the value of raw expression values of *Hba-a1*. The proportion of cells expressing *Hba-a1* among all cells are labelled on the right-top corner. For Visium (probe), *Hba-a2* was plotted because it did not capture transcripts from *Hba-a1.*

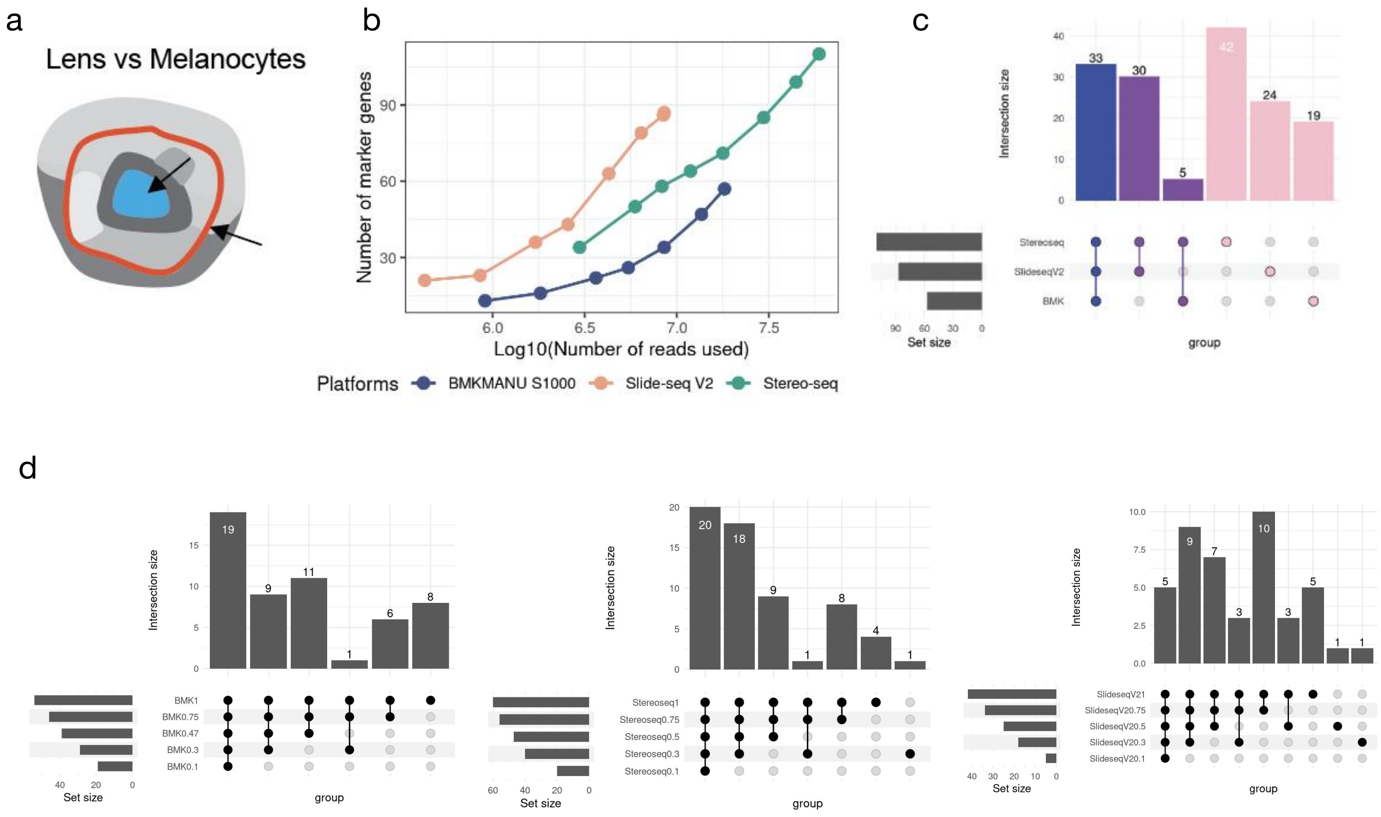

Supplementary Figure 26,
a) An overview of cell states compared in the marker gene detection analysis, with lens and melanocytes highlighted.
b) Number of marker genes detected with different numbers of reads used for each sST method in the comparison between lens and melanocytes.
c) An Upset plot displays the intersection of marker genes obtained by different sST methods using all reads for the lens and melanocytes comparison. Genes shared among all three platforms are denoted in blue, those shared between two platforms are in purple, and uniquely obtained genes are represented in pink.

d) Upset plots displaying the intersection of marker genes obtained by different sST methods using different proportion of reads for the pNR4 and pNR1 comparison.

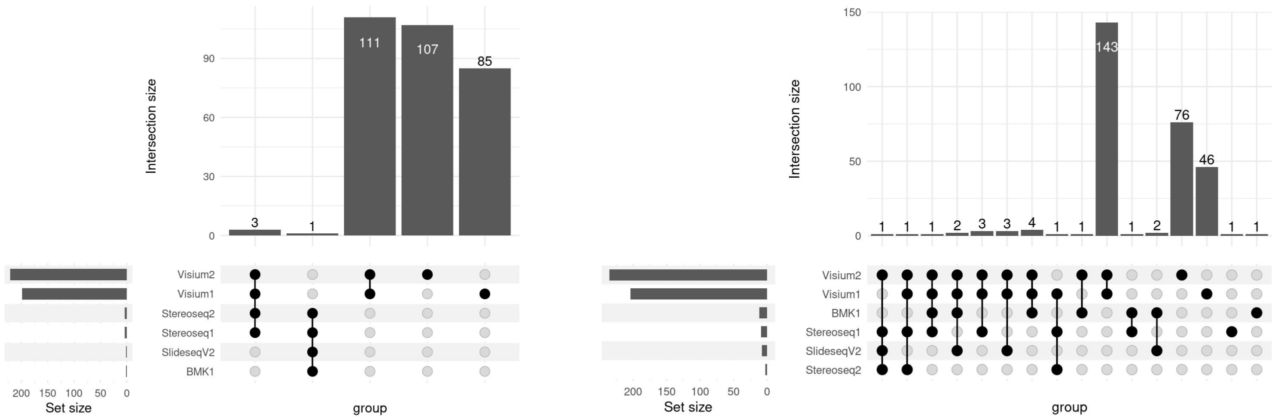

Supplementary Figure 27, Upset plots displaying the intersection of enriched ligand-receptor pairs obtained by different sST methods with results of *cellchat* shown in the left panel, results of *cellphoneDB* shown in the right panel. Visium here represents Visium (polyA).

a

b

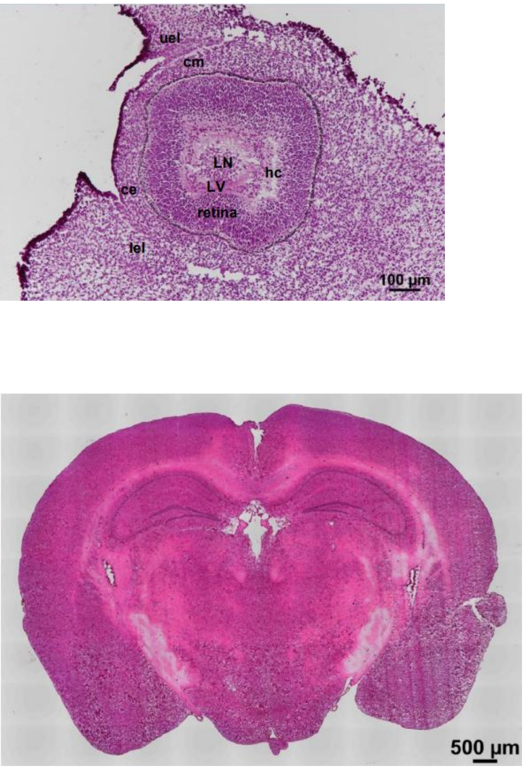

Supplementary Figure 28, The structure of the sequenced cryosections is shown in a) for mouse eye and b) for mouse brain.
