# Supplementary material for: Systematic comparison of sequencing-based spatial transcriptomic methods
Source: Nat Methods. 2024 Jul 4;21(9):1743–54. doi: 10.1038/s41592-024-02325-3 (PMC11399101; doi:10.1038/s41592-024-02325-3)
Supplement: Supplementary file 2 — Reporting Summary [file 41592_2024_2325_MOESM2_ESM.pdf]

nature portfolio

Corresponding author(s): Xiaodong Liu
Luyi Tian

Last updated by author(s): 2024/04/29

Reporting Summary

Nature Portfolio wishes to improve the reproducibility of the work that we publish. This form provides structure for consistency and transparency in reporting. For further information on Nature Portfolio policies, see our Editorial Policies and the Editorial Policy Checklist.

Statistics

For all statistical analyses, confirm that the following items are present in the figure legend, table legend, main text, or Methods section.

n/a	Confirmed	
☐	☒	The exact sample size (*n*) for each experimental group/condition, given as a discrete number and unit of measurement
☐	☒	A statement on whether measurements were taken from distinct samples or whether the same sample was measured repeatedly
☐	☒	The statistical test(s) used AND whether they are one- or two-sided<br>*Only common tests should be described solely by name; describe more complex techniques in the Methods section.*
☒	☐	A description of all covariates tested
☒	☐	A description of any assumptions or corrections, such as tests of normality and adjustment for multiple comparisons
☒	☐	A full description of the statistical parameters including central tendency (e.g. means) or other basic estimates (e.g. regression coefficient) AND variation (e.g. standard deviation) or associated estimates of uncertainty (e.g. confidence intervals)
☐	☒	For null hypothesis testing, the test statistic (e.g. *F*, *t*, *r*) with confidence intervals, effect sizes, degrees of freedom and *P* value noted<br>*Give P values as exact values whenever suitable.*
☒	☐	For Bayesian analysis, information on the choice of priors and Markov chain Monte Carlo settings
☒	☐	For hierarchical and complex designs, identification of the appropriate level for tests and full reporting of outcomes
☒	☐	Estimates of effect sizes (e.g. Cohen's *d*, Pearson's *r*), indicating how they were calculated

Our web collection on statistics for biologists contains articles on many of the points above.

Software and code

Policy information about availability of computer code

Data collection	spaceranger (v2.1.0), BSTMatrix (v2.3.j), Slide-seqV2, SAW (v6.1), scPipe (v2.0.0), STAR (v2.7.10b)
Data analysis	R (v4.3.1), R packages: Seurat (v4.3.0), pheatmap (v1.0.12), ggplot2 (v3.4.2), scPipe (v2.0.0), Cellchat (v1.6.1), DR.SC (v3.3), and PRECAST (v1.6.2);<br>python (v3.11) and python modules: scanpy (v1.4.3), pysam (v0.21.0) cellphoneDB (v4),<br>private code at https://github.com/YOU-k/cadasSTre.

For manuscripts utilizing custom algorithms or software that are central to the research but not yet described in published literature, software must be made available to editors and reviewers. We strongly encourage code deposition in a community repository (e.g. GitHub). See the Nature Portfolio guidelines for submitting code & software for further information.

Data

Policy information about availability of data

All manuscripts must include a data availability statement. This statement should provide the following information, where applicable:

- Accession codes, unique identifiers, or web links for publicly available datasets
- A description of any restrictions on data availability
- For clinical datasets or third party data, please ensure that the statement adheres to our policy

Mouse GRCm39 was used as a reference. Processed data can be downloaded from the National Genome Data Center (https://www.cncb.ac.cn/) under BioProject accession code PRJCA020621. Website is available at https://www.genographix.com.

Human research participants

Policy information about studies involving human research participants and Sex and Gender in Research.

Reporting on sex and gender	n.a.
Population characteristics	n.a.
Recruitment	n.a.
Ethics oversight	n.a.

Note that full information on the approval of the study protocol must also be provided in the manuscript.

Field-specific reporting

Please select the one below that is the best fit for your research. If you are not sure, read the appropriate sections before making your selection.

☒ Life sciences ☐ Behavioural & social sciences ☐ Ecological, evolutionary & environmental sciences

For a reference copy of the document with all sections, see nature.com/documents/nr-reporting-summary-flat.pdf

Life sciences study design

All studies must disclose on these points even when the disclosure is negative.

Sample size	No statistical methods were used to predetermine sample size. The number of embryos and brains reported in the number profiled. the number of embryos and brains analyzed was determined by the quality criteria established for the experiment. For Given the constraints of available resources and budgetary considerations, we opted to conduct one additional replicates. But for some of the datasets, they are of bad quality, so not included in the study.
Data exclusions	We included all embryos and brains collected for each of our datasets.
Replication	All results obtained and reported in this study were reproducible across the replicates examined. Each experiment was replicated 2 times, and each replication was performed independently to maintain the integrity of our study's outcomes.
Randomization	Embryos and brain tissues for every experiment was staged based on morphological features corresponding to the isolation stage. We ensured random allocation to prevent any bias in group assignment. This approach allowed us to accurately assess the biological changes associated with each stage and to draw meaningful conclusions from our experiments.
Blinding	Blinding was not relevant for the experiments. However, our analytical pipeline followed uniform criteria applied to all samples, allowing us to analyze our data in an unbiased manner.

Reporting for specific materials, systems and methods

We require information from authors about some types of materials, experimental systems and methods used in many studies. Here, indicate whether each material, system or method listed is relevant to your study. If you are not sure if a list item applies to your research, read the appropriate section before selecting a response.

Materials & experimental systems

n/a	Involved in the study
☒	☐ Antibodies
☒	☐ Eukaryotic cell lines
☒	☐ Palaeontology and archaeology
☐	☒ Animals and other organisms
☒	☐ Clinical data
☒	☐ Dual use research of concern

Methods

n/a	Involved in the study
☒	☐ ChIP-seq
☒	☐ Flow cytometry
☒	☐ MRI-based neuroimaging

Animals and other research organisms

Policy information about studies involving animals; ARRIVE guidelines recommended for reporting animal research, and Sex and Gender in Research

Laboratory animals	All mice were housed in an SPF facility under a 12/12 hours light/dark cycle, the relative humidity was sustained at 50±10%. Animals were mated at 16:00–17:00, and the vaginal plugs were checked by visual inspection the next morning (8:00–9:00). They are allowed to acclimate to their housing environment for two weeks post arrival. Mouse embryos were collected from pregnant C57BL/6J female mice at embryonic day 12.5 (E12.5). Mouse brain was dissected from 8-week-old C57BL/6J male mice.
Wild animals	The study did not involve wild animals.
Reporting on sex	Findings do not apply to one sex. Male and female embryos were used for analysis.
Field-collected samples	The study did not involve field-collected samples.
Ethics oversight	All relevant procedures involving animal experiments presented in this study are compliant with ethical regulations regarding animal research and were conducted under the approval of the Animal Care and Use committee of the Westlake University (license number AP\#23-111-LXD).

Note that full information on the approval of the study protocol must also be provided in the manuscript.
